# Peptide-binding motifs define the functional architecture of human leukocyte antigens

**DOI:** 10.1186/s13073-026-01825-5

**Published:** 2026-11-12

**Authors:** Pietro Crivello, Mario Hetzel, Julien Racle, David Gfeller, Esteban Arrieta-Bolaños, Katharina Fleischhauer

**Affiliations:** 1 Institute for Experimental Cellular Therapy, University Hospital Essen, Essen, Germany; 2 Department of Oncology UNIL, University of Lausanne, Lausanne, Switzerland; 3 Ludwig Institute for Cancer Research, Lausanne, Switzerland; 4 SIB Swiss Institute of Bioinformatics, Lausanne, Switzerland

**Keywords:** Human leukocyte antigen (HLA), HLA class I and class II, Immunopeptidome, Peptide-binding motifs (PBM), Antigen presentation, HLA polymorphism, Functional HLA diversity

## Abstract

**Background:**

Peptide presentation by polymorphic human leukocyte antigens (HLA) is central to adaptive and innate immunity. HLA polymorphism gives rise to allele-specific peptide-binding motifs (PBM) that shape the immunopeptidomes presented across individuals. However, the global functional architecture imposed by this diversity remains poorly understood.

**Methods:**

Here, we leveraged large-scale immunopeptidomics datasets to perform a comprehensive analysis of PBM across HLA class I (HLA-A, -B, -C) and class II (HLA-DR, -DQ, -DP) molecules. PBM were derived from public resources and *de novo* motif deconvolution of 334,089 class II peptides, followed by hierarchical clustering to define functional groups. We systematically evaluated the association of these groups with immunopeptidome overlap and their relation to established models of HLA functional diversity.

**Results:**

We identified 40 discrete PBM-defined functional groups across 155 class I and 93 class II allotypes. Molecules within the same PBM group showed significantly higher immunopeptidome similarity compared to those across groups. In class II, PBM grouping was further shaped by HLA-DRB3/4/5 paralogues and by α-chain polymorphism in HLA-DQ and -DP, which modulated group assignment of specific β-chains. PBM classification showed only partial concordance with serological antigen groups and evolutionary divergence metrics, but captured differences in peptide repertoires with higher resolution than existing sequence- or epitope-based models. Overall, PBM-based grouping provided a more direct representation of immunopeptidome diversity.

**Conclusions:**

This pan-HLA PBM framework comprehensively maps the functional architecture of antigen presentation and provides a unified platform to functionally classify HLA diversity. By capturing immunopeptidome divergence, PBM groups offer a foundation for improving the interpretation of HLA variation in immunogenetics, transplantation, and immune-mediated diseases.

**Supplementary Information:**

The online version contains supplementary material available at https://doi.org/10.1186/s13073-026-01825-5.

## Background

Human leukocyte antigens (HLA) are central components of the immune system, orchestrating both adaptive and innate immunity. Their main function is to bind and present peptides from intra- and extracellular proteins, providing a mechanism of continuous surveillance of the antigenic landscape. Recognition of peptide-HLA complexes by T-cell receptors (TCR) on T cells or by natural killer (NK) receptors on NK cells triggers cellular responses which are crucial for protection against infections and tumors. At the same time, they are also at the basis of pathogenic processes leading to autoimmune diseases [1] or complications after solid organ or hematopoietic cell transplantation (HCT) [2, 3]. A deeper understanding of HLA biology is therefore pivotal to effectively harness the immune system in different clinical contexts.

HLA molecules are divided into two main classes. While class I (HLA-A, -B, -C) and class II (HLA-DR, -DQ, -DP) molecules share structurally similar peptide-binding domains harboring most of their extensive amino acid variability, they differ in heterodimer composition. Class I molecules consist of a polymorphic α heavy-chain associated with the conserved β2-microgobulin; whereas HLA class II are formed by equimolar α- and β-chains both displaying polymorphism, except for the conserved HLA-DRα. A specific allotype, i.e., the polymorphic HLA molecule, is defined differently for class I and class II. For class I, it is determined by the allele encoding the corresponding heavy chain, while for class II by the combination of alleles encoding the assembled α- and β-chains, in particular for HLA-DQ and -DP. In addition, some but not all HLA-DRB1 genes encoding the HLA-DR β-chain are associated with HLA-DRB3/4/5 paralogues in strong linkage disequilibrium, probably arisen by ancestral duplication. These paralogues further expand the overall HLA-DR diversity. Altogether, while peptide-binding specificity of HLA class I allotypes is primarily determined by the variation encoded within a single polymorphic α heavy-chain, HLA class II peptide-binding properties result from multiple sources of variability, including (i) polymorphism of the β-chain, (ii) αβ-chain pairing in HLA-DQ and HLA-DP, and (iii) for HLA-DR, the additional contribution of the co-expressed HLA-DRB3/4/5 paralogues.

Given the extensive polymorphism and immunological importance of HLA molecules, several models have been proposed to link genetic variability to function, including HLA evolutionary divergence (HED) [4], NK receptor ligand status [5, 6] and HLA-B leader dimorphism [7]. While these models have improved our understanding of HLA biology, they remain mostly locus-restricted or limited by indirect measures of antigen presentation, such as assessment of genetic differences, in vitro cellular assays or clinical transplant outcomes. Thus, the overall functional landscape imposed by HLA diversity remains poorly understood. A more direct way to capture HLA functional divergence is to interrogate the repertoire of naturally presented peptides, the immunopeptidome, linking molecular variability of HLA to the actual peptides being presented. HLA immunopeptidomes can be described by sequence signatures imprinted by each allotype on the presented peptides, and summarized by peptide-binding motifs (PBM). Building on this concept, we previously proposed a PBM-based classification of 122 HLA class I allotypes with 21 distinct PBM groups [8] that informed HCT outcomes [8–11].

Here, we present a pan-HLA classification of PBM groups based on comprehensive immunopeptidome data updates for HLA-A, -B, -C, and on *de-novo* derived motifs for HLA-DR, -DQ and -DP. We specifically explore the contribution of the paralogue β-chain genes HLA-DRB3/4/5 and of α-chain polymorphism in HLA-DQ and HLA-DP, and define the resulting functional architecture of all HLA loci, correlating our findings with existing functional models of HLA diversity. The resulting pan-HLA PBM framework provides a novel benchmark against which to explore the biological and clinical implications of HLA polymorphism.

## Methods

### Data sources

For HLA class I, peptide ligands retrieved from the Major Histocompatibility Complex (MHC) Motif Atlas database were used to calculate PBM as position weight matrices for 123 different allotypes using the motif deconvolution software mixMHCp, as previously described [12]. PBM matrices calculated for 9-mer peptides – the predominant fraction of peptides presented by HLA class I molecules - were used for hierarchical clustering and PBM group definition. For HLA class II, a total of 460,311 peptides were retrieved from three recent immunopeptidomics studies [13–15]. The peptides lists from each publication were merged and filtered to select only peptides assigned to individual HLA class II antigens: a total of 408,718 peptides from 148 heterodimers (53 HLA-DR, 62 HLA-DQ, and 33 HLA-DP) were included in the analysis.

### Motif deconvolution of HLA class II immunopeptidomes

Peptide data from each HLA class II heterodimer were analyzed separately using the software Motif Deconvolution (MoDec v1.2 [16], https://github.com/GfellerLab/MoDec). The analysis was performed with the following parameters: maximum number of motifs up to 5 (--Kmax 5), MHC2 mode enabled (--MHC2) for optimized analysis of HLA class II peptides, number of independent runs set to 10 (--nruns 10), and data output report in HTML format (--makeReport). The optimal number of identified motifs was selected based on the minimum calculated Akaike Information Criterion (AIC), balancing model fitting and complexity, followed by manual inspection and review for quality and consistency. Peptide lists were excluded from downstream analysis if they included low-quality motifs, or motifs not consistent with the analyzed locus, resulting in the exclusion of 55/148 datasets. Overall, 334,089 peptides contributed to PBM determination for 93 HLA class II heterodimers, with motifs spanning a 9-mer core as the minimal binding region recognized by the respective HLA molecule. In case of multiple PBM for an individual heterodimer, primary motifs were defined as the ones associated with the largest number of peptides. The final number of allotypes included in this study and their corresponding peptide counts is reported in Additional file 1: Table S1 and Additional file 2: Table S2.

### Hierarchical clustering and definition of PBM groups

For each HLA locus, position weight matrices of unique or primary PBM were analyzed to define clusters of allotypes with similar peptide-binding preferences (Additional file 1: Fig. S1). Each matrix, reporting the frequency of amino acids across the 9 core-peptide positions, was converted into a numeric vector and pairwise compared using Pearson correlation. Hierarchical clustering was performed using 1 minus Pearson coefficient as distance metric with Ward.D2 linkage method. Clustering results were visualized as circular dendrograms, where main branches defined clusters of allotypes with similar motifs (PBM groups). Group assignments were finalized after visual inspection and direct motif comparison. For visualization in reduced dimensional space, t-distributed Stochastic Neighbor Embedding (t-SNE) [17] was applied to Pearson distance matrix using a perplexity of 5 and 100,000 iterations. In the resulting 2D plots, allotypes were color-coded by PBM group.

PBM classification was extended to all alleles with identical peptide-binding domain (P-group typing level, available at https://hla.alleles.org/pages/wmda/p_groups/), covering a total of 2,488 and 1,687 HLA class I and HLA class II alleles, respectively. Allele frequencies in different populations, as reported by Hurley et al. [18], were used to calculate the cumulative frequency of each PBM group.

### Immunopeptidome overlap analysis

The similarity between immunopeptidomes of HLA allotypes within and across PBM groups was analyzed by pairwise comparisons of peptides presented by each allotype within a given locus. For HLA class I, a total of 301,341 curated peptides were retrieved from the MHC Motif Atlas [12]. For HLA class II, 334,089 peptides were obtained after MoDec clustering (as described above). For each allotype pair, the number of shared peptide sequences was determined, and the overlap quantified using the Dice coefficient, calculated as: 2 x (number of shared peptides)/(sum of peptides for both allotypes). Heatmaps were generated to visualize the degree of immunopeptidome overlap between allotypes from the same or different PBM groups and calculated Dice coefficients between intra- and inter-group comparisons were statistically evaluated using Mann-Whitney U test.

### HLA classification using current models of functional divergence

PBM classification was compared to the following alternative functional groups and models. *Antigen groups.* HLA allotypes included in this study were assigned to antigen groups defined by either their first-field allele names or by corresponding serotypes as defined by Osoegawa and collaborators [19]. A contingency table for each locus was generated to quantify the distribution of alleles across antigen and PBM groups. Statistic associations were evaluated using Fisher’s exact test with Monte Carlo simulation, with effect size quantified by Cramér’s V. Normalized mutual information (NMI) was used to assess shared information between classifications. *HLA evolutionary divergence (HED)*. HED scores were calculated for each pair of different HLA allotypes within each locus using the Pearl script described by Pierini and Lenz [4] (available at https://sourceforge.net/projects/granthamdist/*).* For each allotype pair, HED scores and 1 minus Pearson coefficients of their PBM were compared against the corresponding immunopeptidome overlaps. Spearman correlation coefficients were calculated for both. Additionally, immunopeptidome overlaps were compared for allotype pairs defined within/across PBM groups or with HED higher/lower than locus-specific median. *NK ligand classification*. For HLA class I molecules, HLA allotypes were grouped based on their role as ligands for NK cells defined by the motifs Bw4/Bw6 (amino acids 77–83: N77, I80, L82, R83 for Bw4 and S77, N80, L82, Q83 for Bw6) and C1/C2 (amino acids 77 and 80: S77, N80 for C1 and N77, K80 for C2) shared by some HLA-A/HLA-B and HLA-C allotypes, respectively [5, 6]. Distribution of NK ligand signatures in each HLA class I PBM group was then determined. *HLA-B leader dimorphism*. Each HLA-B allotype was assigned to a B leader group based on the polymorphism at position − 21, methionine (M) or threonine (T), and to their corresponding PBM group. The M/T polymorphism for each HLA-B allele was obtained from the allele alignment available in IMGT/HLA database [20]. Distribution of alleles with either M- or T-variants in the B-leader per each PBM group were visualized using alluvial plot. *G1/G2 groups for HLA-DQ allotypes*. Alpha/beta chain pairing for HLA-DQ heterodimers was shown to be influenced by their polymorphism so that productive combinations of HLA-DQ alpha and beta chains were defined as G1 (DQA1*02/03/04/05/06 with any DQB1*02/03/04) or G2 (DQA1*01 with DQB1*05/06) as previously described [7]. Distribution of PBM groups for heterodimers of the G1/G2 groups was analyzed by cross-tabulation and evaluation of the concordance between the two models. *T-cell epitope (TCE) groups for HLA-DP*. HLA-DP heterodimers were divided into 4 groups using the most recent classification [21]. This classification was compared to the PBM group assignments as by this study using crosstabulation and assessment of concordance between the two models.

### Data visualization and statistical analysis

Data visualization and statistical analyses were performed using either GraphPad PRISM v.9.5.1 (GraphPad Software, Boston, MA, USA), Microsoft Excel 2016 (Microsoft Corporation, Redmond, WA, USA) or RStudio 2022.07.1 (RStudio, PBC, Boston, MA, USA) with R version [4.3.3]. p-values < 0.05 were considered statistically significant. The following R packages were used: *dplyr* and *tidyr* for data manipulation; *vcd* and *DescTools* for Chi-squared test and Cramér’s V computation; *amap*, *dendextend*, *circlize* and *Rtsne* for clustering analysis and dimensionality reduction; *ggplot2*, *ggseqlogo*, *ggalluvial*, *igraph* and *ggraph* for data visualization and plot generation.

## Results

### Defining PBM groups in HLA class I and class II

To establish a comprehensive map of peptide-binding specificities across all major HLA loci, we analyzed PBM of both class I and class II molecules, and defined PBM groups using hierarchical clustering (Additional file 1: Fig. S1).

**Fig. 1:**
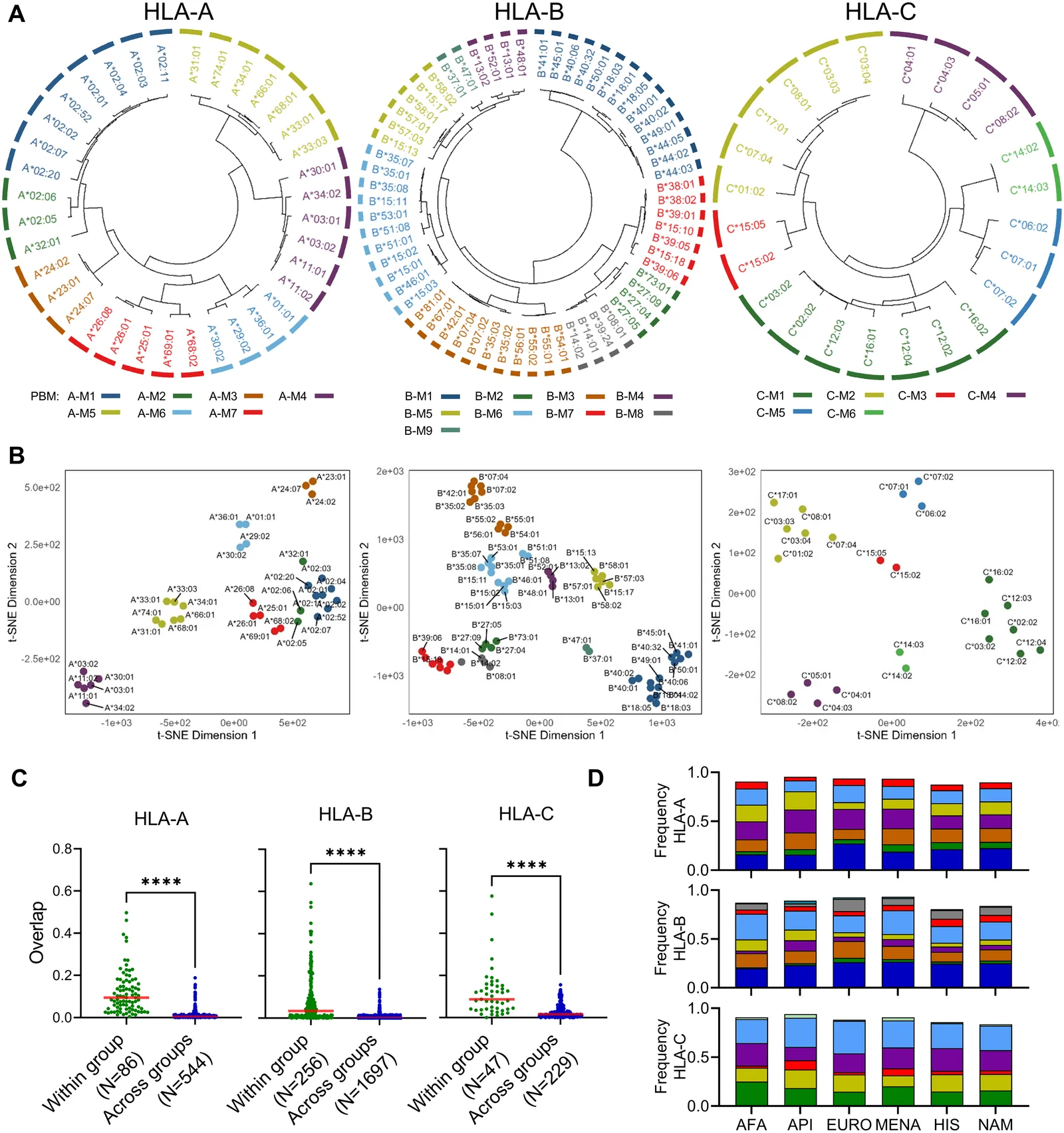
HLA class I PBM groups. **A** Circular dendrograms showing hierarchical clustering of PBM for 123 HLA-A, -B, and -C allotypes retrieved from the MHC Motif Atlas database [12]. Color-codes of alleles encoding the different HLA molecules correspond to the assigned PBM groups, as in the legend. **B** The same HLA class I allotypes with PBM color coding as in panel A, represented in a two-dimensional space following dimensionality reduction with t-SNE. **C** Immunopeptidome overlap assessed by Dice coefficients for allele pairs within the same or across different PBM groups. Statistical comparison by Mann-Whitney test (**** for *p* < 0.0001). **D** Cumulative frequencies of the identified PBM groups across different human populations [18]. PBM group frequencies were calculated as the sum of frequencies for all alleles assigned to each PBM group, including alleles with identical peptide-binding domain. Colors are as in legend to panel A. AFA, African/African American; API, Asian/Pacific Islands; EURO, European/European descent; MENA, Middle East/North Coast of Africa; HIS, South or Central America/Hispanic/Latino; NAM, Native American populations

For class I, PBM of 123 allotypes (36 HLA-A, 63 HLA-B, and 24 HLA-C) were retrieved from the MHC Motif Atlas database [22]. This set comprised 90 allotypes with previously reported PBM group assignments and 33 newly characterized molecules. Altogether, 22 PBM groups were identified (7 HLA-A, 9 HLA-B and 6 HLA-C), of which 21 corresponded to previously reported groups [8] and one was newly defined (Fig. 1A; Table 1, Additional file 1: Fig. S2-S4). Dimensionality reduction using t-SNE supported the consistency of this classification, as allotypes within the same group clustered together in the two-dimensional space (Fig. 1B). In line with their shared motifs, immunopeptidome overlaps were significantly higher for class I allotypes within the same PBM group than allotypes across different PBM groups (Fig. 1C, Additional file 1: Fig. S5). The cumulative allele frequencies corresponding to each PBM group revealed limited variability across populations (Fig. 1D). Together with 32 allotypes previously assigned to PBM groups [8] but not included in the present analysis, the overall PBM classification based on experimental data covered a total of 155 class I molecules.

**Table 1:** PBM classification of HLA-A, -B and -C alleles coding the 123 class I allotypes included in this study

Locus	PBM	*N*. of alleles*	Allele
A	A-M1	8 (213)	02:01, 02:02, 02:03, 02:04, 02:07, 02:11, 02:20, 02:52
	A-M2	3 (66)	02:05, 02:06, 32:01
	A-M3	3 (147)	23:01, 24:02, 24:07
	A-M4	6 (215)	03:01, 03:02, 11:01, 11:02, 30:01, 34:02
	A-M5	7 (180)	31:01, 33:01, 33:03, 34:01, 66:01, 68:01, 74:01
	A-M6	4 (151)	01:01, 29:02, 30:02, 36:01
	A-M7	5 (69)	25:01, 26:01, 26:08, 68:02, 39:01
B	B-M1	14 (276)	18:01, 18:03, 18:05, 40:01, 40:02, 40:06, 40:32, 41:01, 44:02, 44:03, 44:05, 45:01, 49:01, 50:01
	B-M2	4 (66)	27:04, 27:05, 27:09, 73:01
	B-M3	11 (162)	07:02, 07:04, 35:02, 35:03, 42:01, 54:01, 55:01, 55:02, 56:01, 67:01, 81:01
	B-M4	4 (77)	13:01, 13:02, 48:01, 52:01
	B-M5	6 (70)	15:13, 15:17, 57:01, 57:03, 58:01, 58:02
	B-M6	11 (237)	15:01, 15:02, 15:03, 15:11, 35:01, 35:07, 35:08, 46:01, 51:01, 51:08, 53:01
	B-M7	7 (73)	15:10, 15:18, 38:01, 38:02, 39:01, 39:05, 39:06
	B-M8	4 (72)	08:01, 14:01, 14:02, 39:24
	B-M9	2 (26)	37:01, 47:01
C	C-M1	7 (213)	02:02, 03:02, 12:02, 12:03, 12:04, 16:01, 16:02
	C-M2	6 (271)	01:02, 03:03, 03:04, 07:04, 08:01, 17:01
	C-M3	2 (47)	15:02, 15:05
	C-M4	4 (191)	04:01, 04:03, 05:01, 08:02
	C-M5	3 (305)	06:02, 07:01, 07:02
	C-M6	2 (36)	14:02, 14:03

* Numbers of alleles encoding the HLA class I allotypes included in the PBM clustering analysis of this study. Values in brackets represent the total number of alleles classified in each PBM group based on either PBM clustering or sequence identity of the peptide-binding domain to any other classified allele

**Fig. 2:**
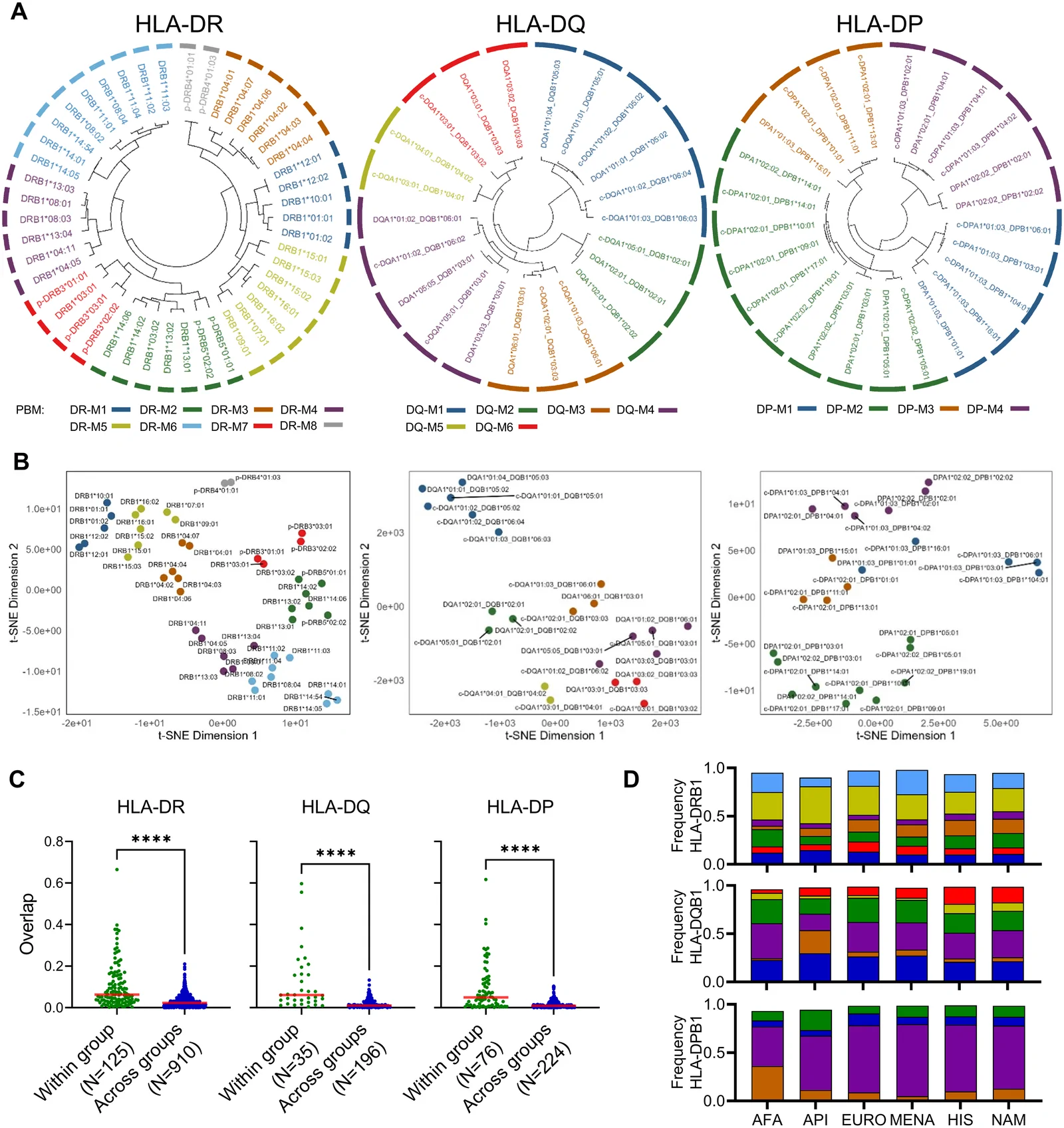
HLA class II PBM groups. **A** Circular dendrograms showing hierarchical clustering of PBM of all 93 HLA-DR, HLA-DQ and HLA-DP heterodimers with available immunopeptidome data (Additional file 1: Table S1 and Additional file 2: Table S2, and Additional file 1: Fig. S6-S7). The corresponding alleles encoding for these molecules are indicated in different colors according to their assigned PBM groups. For HLA-DR, paralogues encoded by DRB3/4/5 were indicated with the prefix “p”. For HLA-DQ and -DP, heterodimers formed by the αβ chains most frequently found in cis in Caucasian populations are indicated with the prefix “c”. **B** The same HLA class II allotypes as in panel A, are represented in a two-dimensional space following dimensionality reduction with t-SNE. Each point representing a different HLA molecule is colored according to the PBM groups in legend to panel A. **C** Immunopeptidome overlap assessed by Dice coefficients for allotype pairs within the same or across different PBM groups. Statistical comparison by the Mann-Whitney test (**** for *p* < 0.0001). **D** Cumulative frequencies of the identified PBM groups in the major HLA class II β chain genes (HLA-DRB1, DQB1 and DPB1) across different human populations [18]. PBM group frequencies were calculated as the sum of the frequencies for all alleles assigned to each PBM group, including alleles with identical peptide-binding domain. For HLA-DQB1 and -DPB1 encoded heterodimers, frequencies were assigned based on the most frequent cis-encoded αβ-heterodimers and their corresponding PBM. Colors are as in legend to panel A. AFA, African/African American; API, Asian/Pacific Islands; EURO, European/European descent; MENA, Middle East/North Coast of Africa; HIS, South or Central America/Hispanic/Latino; NAM, Native American populations

Unlike class I molecules, whose peptide-binding specificity is primarily determined by the variability encoded within their single polymorphic α-chain, class II peptide-binding properties are shaped at multiple levels, including β-chain polymorphism, αβ-chain pairing in HLA-DQ and DP, and contribution of HLA-DRB3/4/5 paralogues for HLA-DR. To capture this additional complexity, we analyzed peptides from an extensive set of class II αβ-chain heterodimers, including molecules carrying the same β-chain paired with different α-chains and HLA-DR heterodimers encoded by DRB3/4/5 paralogues. We collected a total of 460,311 peptides from recent immunopeptidomics studies [13–15], with 334,089 of them specifying peptide-binding motifs for 93 individual αβ-chain heterodimers (46 HLA-DR, 22 HLA-DQ, and 25 HLA-DP). An overview of the immunopeptidome data used for these analyses is provided in Additional file 1: Table S1, Additional file 2: Table S2 and Additional file 1: Fig. S6-S7. Most of the class II allotypes displayed unique PBM, with a few exceptions in HLA-DR and -DP confirming previously reported secondary motifs [14, 23](Additional file 1: Fig. S8). We also identified a so far unreported alternative motif for HLA-DPB1*02 paired with the α-chain HLA-DPA1*02. Only unique or primary motifs were used for PBM clustering analysis across all available class II allotypes, including those encoded by the paralogues DRB3/4/5 for HLA-DR, and both cis- and trans-encoded αβ heterodimers for HLA-DQ and -DP. All molecules were assigned to a total of 18 PBM groups (8 HLA-DR, 6 HLA-DQ, and 4 HLA-DP) (Fig. 2A; Table 2, Additional file 1: Fig. S9-S11). As for class I, class II allotypes within the same PBM group showed similar t-SNE clustering (Fig. 2B), and significantly higher immunopeptidome overlap compared to allotypes across different PBM groups (Fig. 2C, Additional file 1: Fig. S12). The cumulative allele frequencies corresponding to each PBM group of the major β chain loci (HLA-DRB1, -DQB1 and -DPB1), with DQB1 and DPB1 assignments based on their respective cis-encoded αβ-chain combinations, revealed limited variability across populations (Fig. 2D).

**Table 2:** PBM classification of HLA-DRB1, -DRB/3/4/5, -DQB1 and -DPB1 alleles coding the β-chains of the 93 allotypes included in this study

Locus	PBM	*N*. of alleles*	Allele
DRB1	DR-M1	5 (81)	01:01, 01:02, 10:01, 12:01, 12:02
	DR-M2	5 (58)	03:02, 13:01, 13:02, 14:02, 14:06
	DR-M3	6 (63)	04:01, 04:02, 04:03, 04:04, 04:06, 04:07
	DR-M4	6 (40)	04:0, 04:11, 08:01, 08:03, 13:03, 13:04
	DR-M5	7 (116)	07:01, 09:01, 15:01, 15:02, 15:03, 16:01, 16:02
	DR-M6	9 (71)	08:02, 08:04, 11:01, 11:02, 11:03, 11:04, 14:01, 14:05, 14:54
	DR-M7	1 (26)	03:01
DRB3	DR-M7	3 (127)	01:01, 02:02, 03:01
DRB4	DR-M8	2 (53)	01:01, 01:03
DRB5	DR-M2	2 (39)	01:01, 02:02
DQB1^2^	DQ-M1	5 (224)	05:01, 05:02, 05:03, 06:03, 06:04
	DQ-M2	2 (101)	02:01, 02:02
	DQ-M3	2 (66)	03:03, 06:01
	DQ-M4	2 (186)	03:01, 06:02
	DQ-M5	2 (46)	04:01, 04:02
	DQ-M6	1 (63)	03:02
DPB1^2^	DP-M1	4 (65)	03:01, 06:01, 16:01, 104:01
	DP-M2	6 (109)	05:01, 09:01, 10:01, 14:01, 17:01, 19:01
	DP-M3	4 (91)	01:01, 11:01, 13:01, 15:01
	DP-M4	4 (281)	02:01, 02:02, 04:01, 04:02

* Numbers of the HLA class II β-chain coding alleles of the 93 allotypes included in the PBM clustering analysis of this study. Values in brackets represent the total number of β-chain alleles classified in each PBM group based on either PBM clustering or sequence identity of the peptide-binding domain to any other classified allele. ^2^ For HLA-DQB1 and -DPB1 alleles, PBM groups were assigned based on the cis-encoded αβ-heterodimers

To further broaden coverage of the PBM classification, we also included alleles encoding peptide-binding domain amino acid sequences identical to those of the analyzed allotypes, under the assumption that they share overlapping peptide-binding characteristics (so-called P_group alleles). This resulted in the classification of 3,009 additional class I (991 for HLA-A, 979 for HLA-B, and 1,039 for HLA-C) and 1,828 class II alleles (416 for HLA-DRB1, 212 for HLA-DRB3/4/5, 672 for HLA-DQB1, and 528 for HLA-DPB1) (Additional file 3: Table S3), reaching an overall allele frequency coverage higher than 80% in each locus across different human populations (0.87–0.95 for HLA-A, 0.82–0.93 for HLA-B, 0.81–0.94 for HLA-C, 0.90–0.98 for HLA-DRB1, 0.96–0.99 for HLA-DQB1, and 0.93–0.99 for HLA-DPB1).

### Contribution of HLA-DRB3/4/5 to the functional diversity of HLA-DR

Certain HLA-DRB1 alleles are in tight linkage disequilibrium with their paralogues HLA-DRB3, -DRB4 or -DRB5 (DRB3/4/5). The presence of these paralogues increases the heterogeneity of αβ-chain heterodimers on the cell surface, although linkage restricts which DRB1 ~ DRB3/4/5 haplotype combinations naturally occur. Cells heterozygous for two HLA-DRB1 alleles that are each linked with a DRB3/4/5 paralogue will assemble up to four different αβ-chain heterodimers. Although DRB3/4/5 are generally considered to be expressed at lower levels than HLA-DRB1, recent transcriptomic and proteomic data indicate that their expression is not negligible and can reach levels comparable to that of other major HLA loci [24, 25]. We confirmed this in particular for HLA-DRB3, which reached protein expression levels comparable to DRB1 based on the analysis of a published proteome compendium of immune cells [26] (Additional file 1: Fig. S13).

**Fig. 3:**
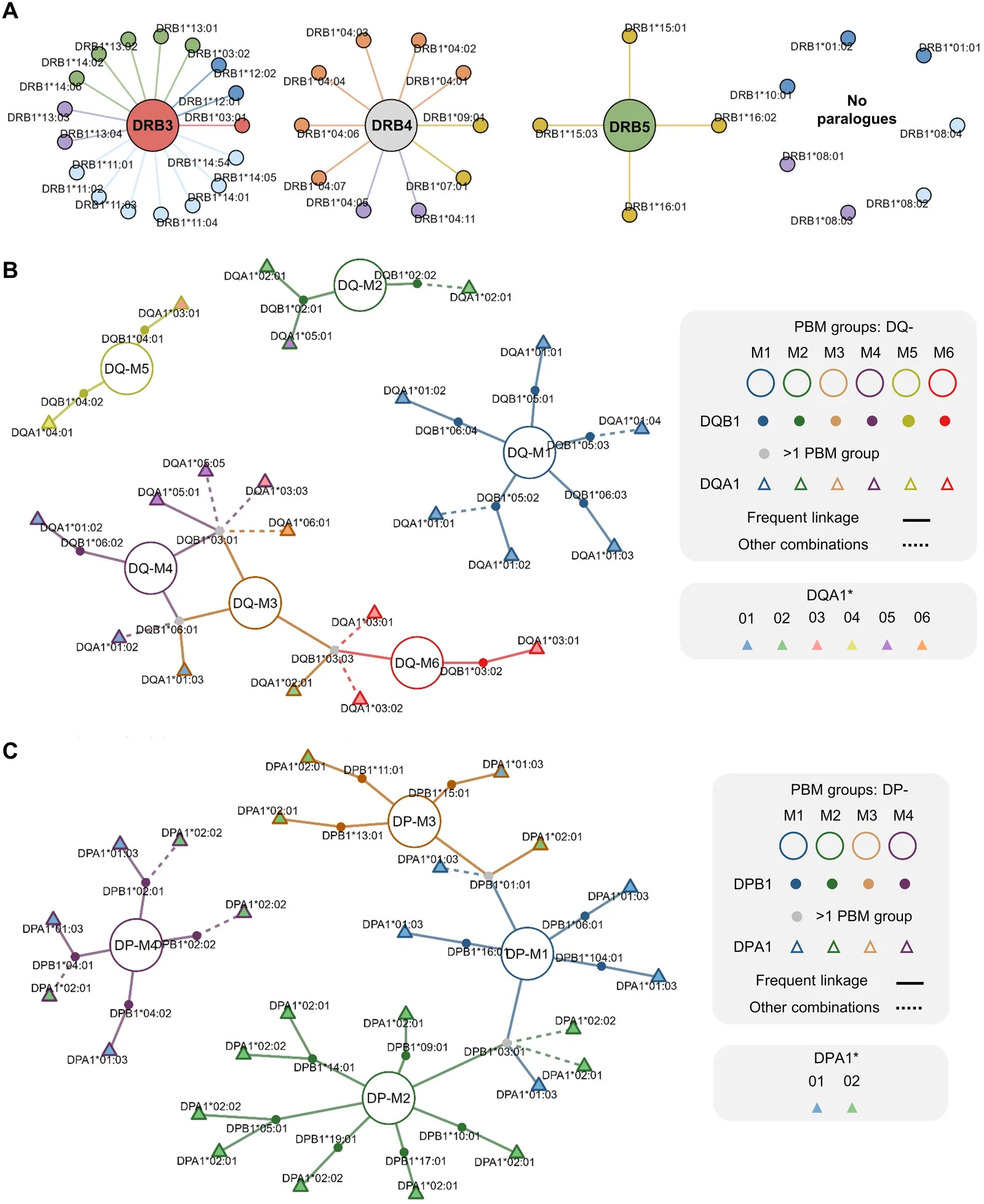
Contribution of HLA-DRB3/4/5 paralogues and HLA-DQ/DPαβ chain pairing to class II PBM diversity. **A** Radial network plots showing the contribution of HLA-DRB3, DRB4 and DRB5 paralogues to HLA-DR PBM diversity through their linkage with the indicated HLA-DRB1-encoded allotypes (PBM group assignment is indicated by colors as in legend to Fig. 2A). Matching colors between HLA-DRB3/4/5 and the connected HLA-DRB1 indicate shared PBM classification. **B** Network representation of HLA-DQA1 ~ DQB1 combinations and their assigned PBM groups. Main nodes represent identified PBM groups connected to corresponding HLA-DQB1 (circle nodes) and DQA1 (triangles) alleles. Edge colors reflect the PBM group assignment of each αβ-combination. HLA-DQB1 nodes are colored according to their PBM group assignment, or shown in grey when the same β-chain contributes to more than one PBM group, depending on the paired α-chain. For HLA-DQA1 nodes, border colors reflect the PBM group assignment of the αβ heterodimer, whereas fill colors correspond to their first-field allele designation. Solid lines label combinations commonly found in linkage disequilibrium. **C** Network representation of HLA-DPA1 ~ DPB1 pairs and their PBM groups following the same scheme described in panel B for HLA-DQ heterodimers

PBM clustering of DRB3/4/5-derived heterodimers revealed distinct contributions to the overall peptide-binding diversity of HLA-DR. DRB3 heterodimers were found to fall into the same PBM group as one of their linked DRB1 paralogues, HLA-DRB1*03:01, but none of the others. Consequently, DRB1 ~ DRB3 haplotypes can either contribute a single PBM group, as observed for HLA-DRB1*03:01 ~ DRB3, or two distinct ones in other haplotypes (Fig. 3A, and Additional file 1: Fig. S9). In contrast, DRB5 heterodimers clustered into a PBM group shared by a number of DRB1-encoded allotypes, but none of them were among those in linkage disequilibrium with DRB5. Similarly, DRB4 heterodimers formed a distinct PBM group that was not shared by any other HLA-DR allotype, including those encoded by any of its linked DRB1 alleles (Figs. 2A and 3A and Additional file 1: Fig. S9). Therefore, DRB1 ~ DRB4 and DRB1 ~ DRB5 haplotypes expand the overall HLA-DR repertoire by adding an additional PBM specificity distinct from the one encoded by the associated DRB1 allotype.

### HLA-DQ and DP αβ-chain pairing modulates PBM group assignment

For HLA-DQ and HLA-DP, polymorphism of the α-chain can lead to the formation of up to four different heterodimers through the combination of two different α-chains with two different β-chains encoded on each chromosome of heterozygous individuals. Although each HLA-DQB1 and HLA-DPB1 allele is tightly linked to a specific *cis*-encoded α-chain, dimerization can occur with some *trans*-encoded HLA-DQA1 or -DPA1 in heterozygous cells. This additional layer of heterodimer variability impacted PBM group assignments in some cases, where the same β-chain was associated with different PBM groups depending on its paired α-chain. In particular, this effect was evident for HLA-DQB1*03:01, 03:03, 06:01 (Fig. 3B), and for HLA-DPB1*03:01 and 01:01 allotypes (Fig. 3C), where PBM group assignment was influenced by the associated α-chain. These allotypes represent illustrative examples of the modulatory effect of α-chain polymorphism on PBM classification, the full extent of which will require further investigation as additional data become available. Notably, PBM group changes could be prompted by closely related α-chains such as DQA1*01:02 vs. 01:03 and DPA1*01:03 and 02:01, differing for only 5 and 7 amino acids, respectively.

### PBM groups and other models of functional HLA diversity

HLA polymorphism and the resulting variability of presented peptides has been described by different functional models, including epitopes defining serological antibody [27] or NK cell receptor binding [5, 6], as well as, more recently, HLA evolutionary divergence (HED) to capture differences in the presented peptide repertoire between allotype pairs [4]. It has also been suggested that the breadth of the immunopeptidome could be influenced by the degree of tapasin dependency of class I molecules [28]. Variability in HLA-B leader sequences, tightly linked to exon 2 and 3 polymorphism, has been suggested as a relevant modulator of T- and NK-cell immunity [29, 30] but the relationship between B-leader genotype and HLA-B immunopeptidomes has not been explored. Finally, both HLA-DQ and HLA-DP molecules have been clustered into different functional groups relevant in allogeneic HCT, according to the ability of αβ-chain pairing and alloreactive T-cell cross-reactivity, respectively [21, 31–33].

**Fig. 4:**
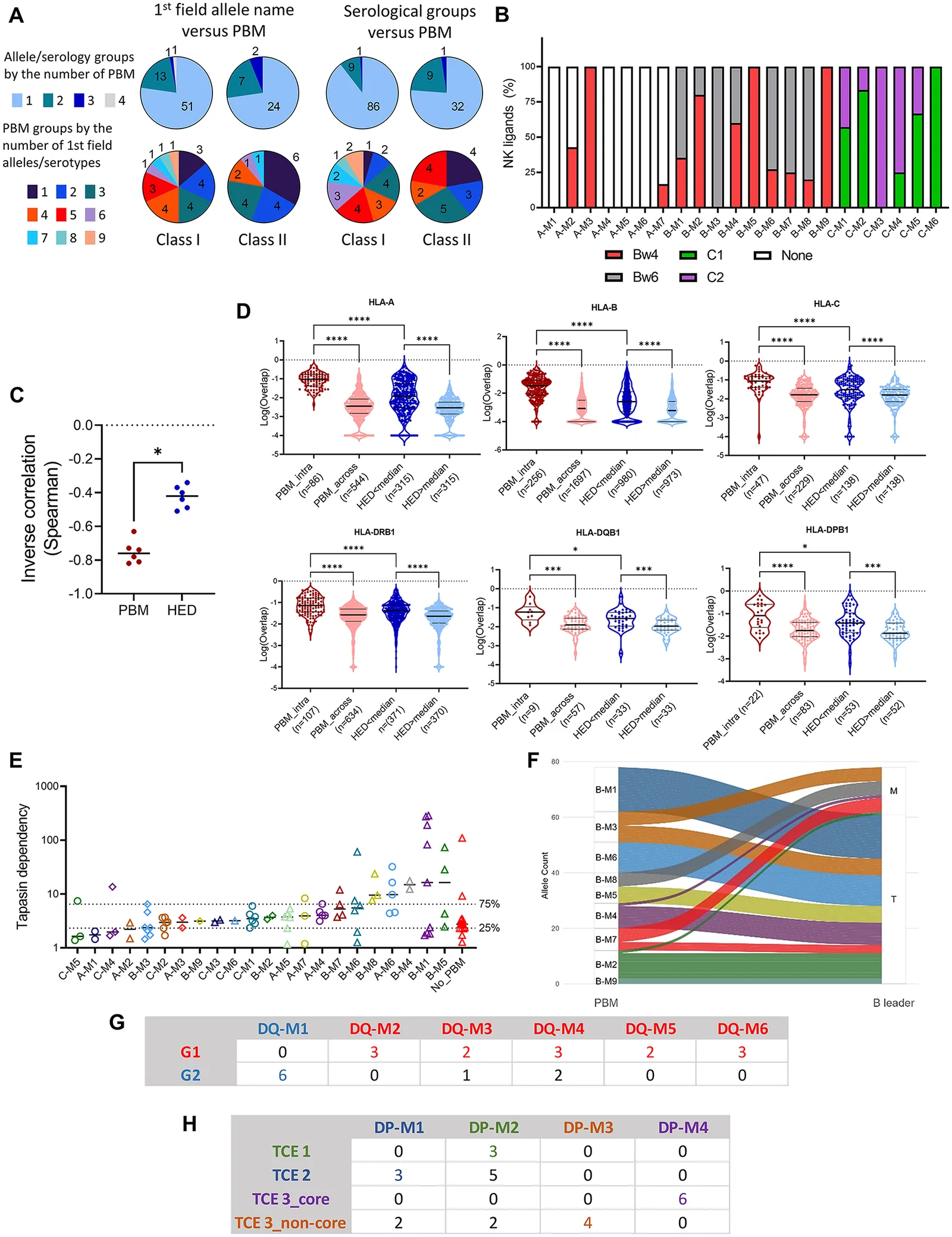
Comparison of PBM groups with other functional measures of HLA diversity. **A** Distribution of HLA first-field allele groups and serological antigen groups defined by Osoegawa et al. [19] by the number of PBM groups within them, and vice versa. For both class I and class II, top pie charts show the number of first-field allele (left) and serological types (on the right) that include one or more PBM groups, as indicated in the legend. Similarly, bottom pie charts show the number of PBM groups including one or more allele/serotype groups as in the legend. **B** PBM groups and class I NK ligand epitopes. Shown is the fraction of HLA-A, -B, and -C allotypes with NK ligand epitopes (Bw4, C1 and C2) or absence of them (Bw6 or none) in each class I PBM group. **C**,** D** PBM groups and HED. **C** Spearman correlation coefficients for all 6 loci were calculated after plotting immunopeptidome overlaps of allotype pairs against either the distance (1 - Pearson coefficient) of the corresponding PBM in hierarchical clustering, or against the calculated HED. Correlation plots are reported in Additional file 1: Fig. S15. The Wilcoxon test was used to compare the two groups, with PBM distances exhibiting a significantly stronger inverse correlation than HED (* for *p* < 0.05). **D** Violin plots depicting the immunopeptidome overlaps between allotype pairs after stratification either by PBM group assignment (within versus across PBM groups) or by the median HED score. The Kruskal-Wallis test was used to compare the different groups. ** and **** indicate *p* < 0.01 and *p* < 0.0001, respectively; ns for not significant. **E** Tapasin dependency scores as described by Bashirova et al. [28] are plotted for each HLA class I allotype in the different HLA class I PBM groups ordered by ascending median. Allotypes with no group assignment in this study are included as “No_PBM”. Dotted lines indicate 1st (25%) and 3rd (75%) quartile cut-offs. **F** Alluvial plot showing the distribution of HLA-B allotypes in different PBM groups (colors as in Fig. 2A), and the corresponding B leader polymorphism - Methionine (M) or Threonine (T) at position − 21. **G**-**H** Contingency tables of HLA-DQ (G) and HLA-DP (H) heterodimers stratified by their PBM groups and assignment to either HLA-DQ G1/G2 groups [32] or HLA-DP TCE groups [21], respectively. Allotypes showing comparable classification across the two systems are highlighted in colors

In order to compare these traditional models of functional HLA variability with the established map of PBM groups, we performed systematic side-by-side comparisons. We first assessed whether PBM classification aligns with first-field HLA allele typing and serological antigen groups [19] (Additional file 1: Fig. S14 and S15). Across all loci, PBM classification showed a strong but incomplete concordance with both allotype groupings, displaying Cramér’s V coefficients of 0.80–0.92 against allele types and 0.76–0.98 against serological groups (all comparisons were statistically significant except for HLA-DPB1 serology). Normalized mutual information (NMI) scores were instead consistently lower (0.52–0.79), reflecting only partial overlap between PBM groups and the other classifications. In particular, most of the first-field alleles and serotypes were assigned to a single PBM group, but a subset of them − 24/98 allele types (e.g. HLA-A*68, -B*15, -DRB1*04 and -DQB1*03) and 20/138 serological groups (e.g. A-6801, B-3501, DR-0801 and DQ6) - were split across multiple groups. In contrast, most PBM groups included allotypes from more than one allele (31/40) or serological type (35/40) (Fig. 4A). These results indicate that PBM groups capture a different functional structure within HLA allotypes not fully represented by first-field typing or serology. Also, when confining the analyses to the dimorphic BW4/BW6, C1/C2 epitopes which define NK-cell receptor binding, we found that most PBM groups comprise allotypes carrying either of the two epitopes (Fig. 4B).

Next, we compared PBM groups with HED, a sequence-based metric that quantifies the amino acid divergence between HLA alleles, and therefore could potentially act as an indirect measure of immunopeptidome divergence [4]. Both HED scores and PBM distances from the clustering described above, calculated for all allele pairs within each locus, showed significant inverse correlation with immunopeptidome overlaps (Additional file 1: Fig. S16). However, the PBM model showed a stronger negative correlation with significantly lower Spearman’s coefficients than those observed for HED (Fig. 4C). To further support this observation, we stratified each pair combination based on their PBM status (within or across PBM group) and HED (above or below the locus-specific median, often used as cutoff to discriminate high versus low HED scores). Both models revealed significant differences in immunopeptidome overlap between conditions, but PBM grouping provided a clearer separation of allotype pairs with high versus low overlap than HED (Fig. 4D).

HLA class I allotypes have been reported to have differential dependency on tapasin-mediated peptide loading, affecting the presented peptide repertoire and therefore representing an additional way of capturing peptide diversity [28]. In this context, we observed that class I allotypes within the same PBM group displayed similar levels of tapasin-dependency, although some variability was present within certain groups (Fig. 4E). This exploratory analysis suggests that PBM similarity and tapasin-dependency may capture related yet distinct and complementary aspects of HLA class I peptide presentation.

We also investigated the hypothesis that B-leader polymorphism, i.e., the presence of methionine (M) or threonine (T) at position − 21 of HLA-B molecules, might be a proxy of PBM groups, given the strong LD between B-leader status and amino acid sequence variability in the peptide antigen binding groove [34]. 4/10 HLA-B PBM groups contained allotypes with both M and T leaders, demonstrating that the two measures are not complete surrogates of each other (Fig. 4F).

Finally, PBM groups were compared to functional models in HLA-DQ and HLA-DP. For HLA-DQ, the G1/G2 model distinguishes α- and β-chain *cis*-group pairings leading to stable and functional HLA-DQ heterodimers, from unstable *trans*-group pairings [33]. Consistent with previous reports [14, 35], we observed a limited contribution of *trans*-group heterodimers to the overall HLA-DQ immunopeptidome (Additional file 1: Table S1). In addition, PBM grouping revealed considerable heterogeneity within both the G1 and the G2 group, with HLA-DQ allotypes belonging to five (G1) or three (G2) distinct PBM groups (Fig. 4G). For HLA-DP, the T-cell epitope (TCE) model divides HLA-DP allotypes into four functional groups (TCE-1, TCE-2, TCE-3 core and non-core) with diverging immunopeptidomes and immunogenicity [21, 36]. Comparison of PBM groups with the TCE-based classification showed partial overlap: 16/25 HLA-DP allotypes were assigned to corresponding functional groups by both approaches (Figs. 3C and 4H). However, some PBM groups included HLA-DP allotypes belonging to different TCE categories. Thus, while there is substantial agreement between the two frameworks, the correspondence is not complete.

## Discussion

In this study, we present a comprehensive functional map of HLA class I and class II allotypes generated by leveraging extensive immunopeptidomics datasets and state-of-the-art bioinformatic approaches. We defined 40 PBM groups that effectively capture the characteristics of immunopeptidomes presented within each locus, with a high allele coverage in worldwide populations. PBM groups also provided novel molecular insights especially into HLA class II antigen presentation, highlighting the functional impact of paralogue β-chain genes (DRB3/4/5) and polymorphic α-chain variants. The resulting map not only aligns with established functional models, but also provides a higher resolution framework to interpret the functional consequences of HLA polymorphism and its implications in different clinical settings, including immunogenetics, transplantation, and immunotherapy.

PBM groups capture immunopeptidome diversity of HLA allotypes, representing a novel framework for their functional classification. The conceptually closest model to characterize peptide diversity between HLA molecules is the HED metric [4], which we showed to be overlapping but less granular than PBM groups. Interestingly, we found a partial but significant overlap between PBM and both serological antigen groups and first-field genotypes, which mirror structural epitopes in the HLA molecule itself rather than the presented peptides. Nevertheless, a substantial fraction of first-field allele groups and serotypes tested over all 6 HLA loci were heterogeneous in their PBM group assignments, while most of the PBM groups included allotypes from multiple allelic and serological groups, suggesting that the two measures are not direct surrogates, but rather complementary. Interestingly, this partial overlap between antigenic and PBM groups also translates into limited correspondence in matching at those levels between patients and donors in HCT [37]. Similarly, we previously compared PBM groups with the established class I supertypes [38, 39] and observed only partial correspondence between the two classifications, with several allotypes differing in their assignment by the two approaches [8]. These differences likely reflect the fact that PBM groups are derived from large-scale experimentally determined endogenously presented immunopeptidomes, enabling a more comprehensive characterization of functional peptide-binding similarities among class I allotypes. Similarly, PBM groups show partial but incomplete overlaps with other functional models studied, including tapasin-dependency, HLA-B leader status and NK receptor binding, as well as the HLA-DQ G1/G2 and HLA-DP TCE models, all of which have been shown to be relevant in different clinical settings such as allogeneic HCT or response to infection. The robust and comprehensive map of HLA functional diversity by PBM groups provided in this study provides a new benchmark against which to test these models, to unravel differences and complementary aspects.

Expanding PBM classification to HLA class II heterodimers provided important insights into the contribution of both α- and β-chain polymorphism in shaping peptide-binding characteristics and immunopeptidome diversity. This is especially relevant for HLA-DQ and HLA-DP molecules, where α-chain variants have been shown to affect heterodimer stability [33] and T-cell recognition [40], respectively. Our data support a limited role of G1/G2 *trans*-heterodimers for the immunopeptidome landscape of HLA-DQ [14, 35], and confirm previous reports of reverse binding motifs in HLA-DP, primarily associated with heterodimers containing the α-chain HLA-DPA1*02 [14, 23]. We also observed an important contribution in the immunopeptidome landscape of HLA-DR by heterodimers containing the paralogue β-chains DRB3/4/5 [41], which frequently increased the number of PBM groups displayed in a single individual. Based on modern transcriptomics and proteomics data, we show that, contrarily to previous dogma, HLA-DRB3 appears to be expressed at similarly high levels as HLA-DRB1 across different antigen presenting cells, suggesting a functional role for the associated increased PBM diversity. From an evolutionary standpoint, it is interesting to note that HLA-DRB3 falls into the same PBM group as HLA-DRB1*03:01, arguing in favor of common ancestry and similar function [42]. HLA-DRB1*03:01 is part of the frequent HLA-A1, -B8, -DR3 haplotype including HLA-DRB3, highlighting the frequent co-occurrence of these molecules in human populations. In contrast, the PBM groups of the other two paralogues HLA-DRB4/5 were distinct from their associated HLA-DRB1 specificities and showed lower expression levels compared to HLA-DRB3, suggesting functional differentiation that may reflect divergent evolutionary trajectories.

The proposed pan-HLA classification holds important translational potential. We have previously shown that in allogeneic HCT, PBM groups informs patient-donor compatibility by predicting the risk of T-cell alloreactivity and consequently, graft rejection or graft-versus-host disease. While models such as TCE matching for HLA-DP and G1/G2 groups for HLA-DQ have been successfully employed [32, 43], PBM groups offer a refined and comprehensive framework to extend predictions across all HLA loci, especially aiding risk stratification in case of multiple HLA mismatches [44–46]. Beyond transplantation, PBM groups may help generalizing HLA allele associations with specific clinical conditions, such as autoimmune diseases or response to infection, as shared peptide-binding characteristics might lead to similar clinical risk and phenotypes. They may also support vaccine design targeting epitopes relevant across PBM groups, broadening applicability beyond single alleles. In addition, the extensive allele coverage achieved in the present PBM classification provides a foundation for future studies investigating the distribution of PBM-defined functional groups across human populations with distinct demographic histories, including geographically isolated or indigenous populations. Such studies will determine whether the relatively conserved distribution of PBM groups in the populations analyzed here is maintained globally or whether population-specific patterns of functional diversity exist. Altogether, the pan-HLA PBM framework provided here will enable future studies to test these exciting hypotheses.

Despite these strengths, the study has a number of limitations. Quality and completeness of immunopeptidomics data vary across HLA allotypes, with common allotypes better characterized than rare ones. This can influence PBM accuracy and confidence of group assignment, which are expected to improve as additional immunopeptidomics data become available. In this context, it should be noted that the refined HLA class I PBM assignments we present here, including PBM data from 33 previously uncharacterized allotypes, resulted in some substantial differences, including the shift of 23 alleles from one PBM group to another, and the identification of a new PBM group in HLA-C. As additional data become available, PBM group assignment may be extended to currently uncharacterized HLA alleles. Moreover, integration with sequence-based peptide-binding predictions and machine-learning models trained on large experimental datasets could provide complementary strategies to predict functional classification beyond experimentally characterized molecules. Another limitation is represented by the focus on peptide-binding features of HLA molecules, which misses factors influencing peptide immunogenicity, such as post-translational modification, peptide editing by tapasin or HLA-DM, and variable expression influenced by tissue specificity or inflammation. Structural characteristics outside the peptide-binding groove, potentially affecting interactions with T-cell and NK receptors, are also not accounted for. Future integration of these factors along with TCR repertoire data and a deeper knowledge of HLA-receptor interactions could enhance PBM resolution and its predictive value.

## Conclusions

In summary, we present a high-resolution functional map of HLA molecules based on PBM groups that capture peptide-binding similarities across allotypes. This classification will aid our interpretation of HLA function in different immunological and clinical contexts, with broad potential applications. Serving as a functional benchmark, PBM groups can enable the development of integrative models that incorporate multiple layers of HLA biology, for the improvement of our understanding of HLA function and its impact on human health.

## Supplementary Information

Below is the link to the electronic supplementary material.

Supplementary Material 1

Supplementary Material 2

Supplementary Material 3

## Data Availability

Peptide datasets and position weight matrices for HLA class I were obtained from the MHC Motif Atlas (http://mhcmotifatlas.org/class, DOI: https://doi.org/10.1093/nar/gkac965) [12]. HLA class II peptide datasets were retrieved from the supplementary materials provided in the studies by Stražar et al. (DOI: https://doi.org/10.1016/j.immuni.2023.05.009) [13] and Racle et al. (DOI: https://doi.org/10.1016/j.immuni.2023.03.009) [14], and from the NetMHCIIpan-4.3 webserver (https://services.healthtech.dtu.dk/services/NetMHCIIpan-4.3/, DOI: https://doi.org/10.1126/sciadv.adj6367) [15]. All PBM group assignments generated in the study are provided in the manuscript and its supplementary tables. The R scripts used for the analyses are publicly available on GitHub and archived on Zenodo [47, 48].

## Abbreviations

HLA: Human Leukocyte Antigen
PBM: Peptide-Binding Motif
TCR: T-Cell Receptor
NK: Natural Killer
HCT: Hematopoietic Cell Transplantation
HED: HLA Evolutionary Divergence
t-SNE: t-distributed Stochastic Neighbor Embedding
AIC: Akaike Information Criterion
NMI: Normalized Mutual Information
TCE: T-cell epitope groups of HLA-DPB1
