# Supplementary material for: Peptide-binding motifs define the functional architecture of human leukocyte antigens
Source: Genome Med. 2026 Nov 12;18:178. doi: 10.1186/s13073-026-01825-5 (PMC13769151; doi:10.1186/s13073-026-01825-5)
Supplement: Supplementary file 1 [file 13073_2026_1825_MOESM1_ESM.pdf]

Supplementary Information for

Peptide-binding motifs define the functional architecture of human leukocyte antigens

Pietro Crivello[1]*, Mario Hetzel[1], Julien Racle[2,3,4], David Gfeller[2,3,4], Esteban Arrieta-Bolaños[1], Katharina Fleischhauer[1]

[1] Institute for Experimental Cellular Therapy, University Hospital Essen, Essen, Germany

[2] Department of Oncology UNIL, University of Lausanne, Lausanne, Switzerland

[3] Ludwig Institute for Cancer Research, Lausanne, Switzerland

[4] SIB Swiss Institute of Bioinformatics, Lausanne, Switzerland

* Correspondence to:

Pietro Crivello

Email: pietro.crivello@uk-essen.de

This PDF file includes:

Tables S1
Figs. S1 to S16

Table S2 and S3 are provided as separate Excel files (Additional file 2 and 3).

Table S1. Overview of the immunopeptidomics data used for PBM clustering of HLA class II allotypes.

Locus	N. of heterodimers*	Clustered peptides Median (range)
HLA-DR	46	4,066 (621-19,761)
DRB1	39	4,189 (621-19,761)
DRB3	3	4,039 (3,305-4,063)
DRB4	2	3,705 (3,317-4,094)
DRB5	2	2,961 (1,034-4,888)
HLA-DQ	22	3,366 (757-12,315)
DQA1*01 cis-dimers (G2)	9	3,288 (757-12,315)
DQA1*02/03/04 cis-dimers (G1)	13	3,469 (866-9,844)
G1G2 trans-dimers	0	0
HLA-DP	25	8,192 (176-16,505)
DPA1*01 cis-dimers	8	8,487 (346-13,434)
DPA1*02 cis-dimers	11	10,548 (1,273-16,505)
DPA_DPB trans-dimers	6	1,730 (176-4,786)

* The complete list of HLA class II heterodimers included in the study is provided in **Additional file 2: Table S2**.

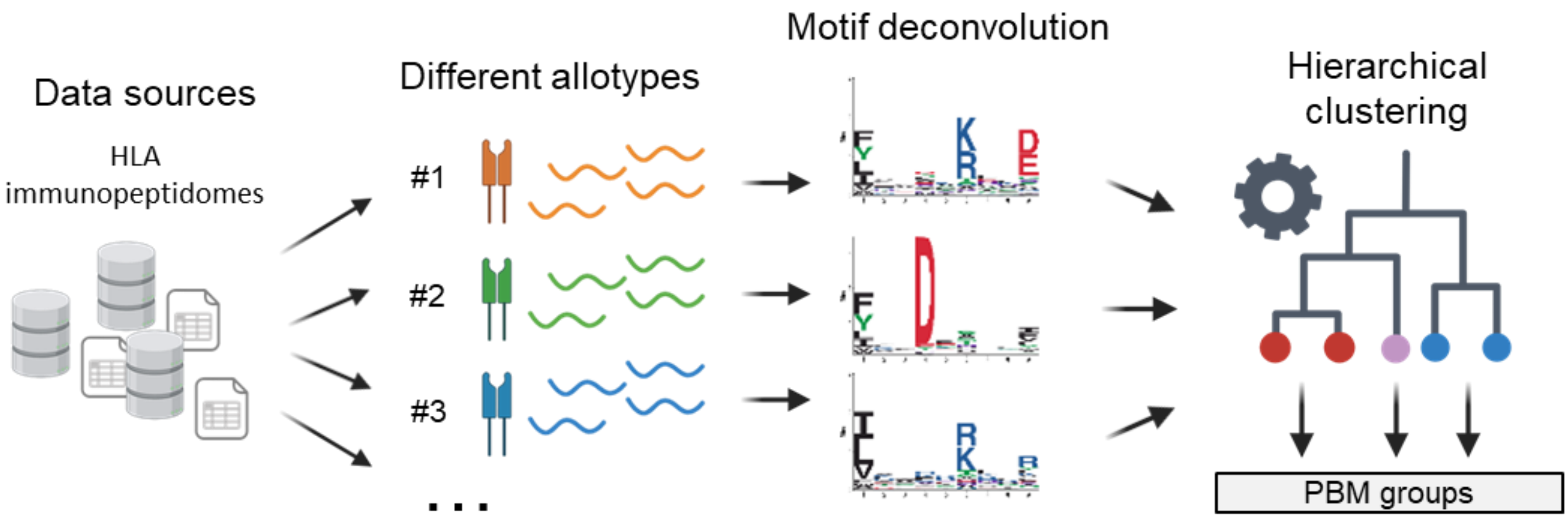

Fig. S1. Workflow for PBM-based functional classification of HLA molecules. Schematic overview of the analysis pipeline used to define PBM groups within HLA loci. The workflow consists of three main steps. 1) Data retrieval: immunopeptidomics datasets are collected, integrated and filtered for peptides assigned to individual HLA allotypes. 2) Motif deconvolution: peptides from each allotype are analyzed to derive position weight matrices that quantify amino acid preferences at each position of a 9-mer peptide core. These matrices represent the PBM of each molecule and can be visualized as sequence logos. 3) PBM grouping: PBM matrices are quantitatively compared using hierarchical clustering to identify clusters of allotypes with similar PBM, named PBM groups. For HLA class II, immunopeptidomics data were collected from three recent studies[13-15] and used for PBM determination in step 2. For HLA class I, PBM were calculated from the curated list of ligands included in the MHC Motif Atlas database[12].

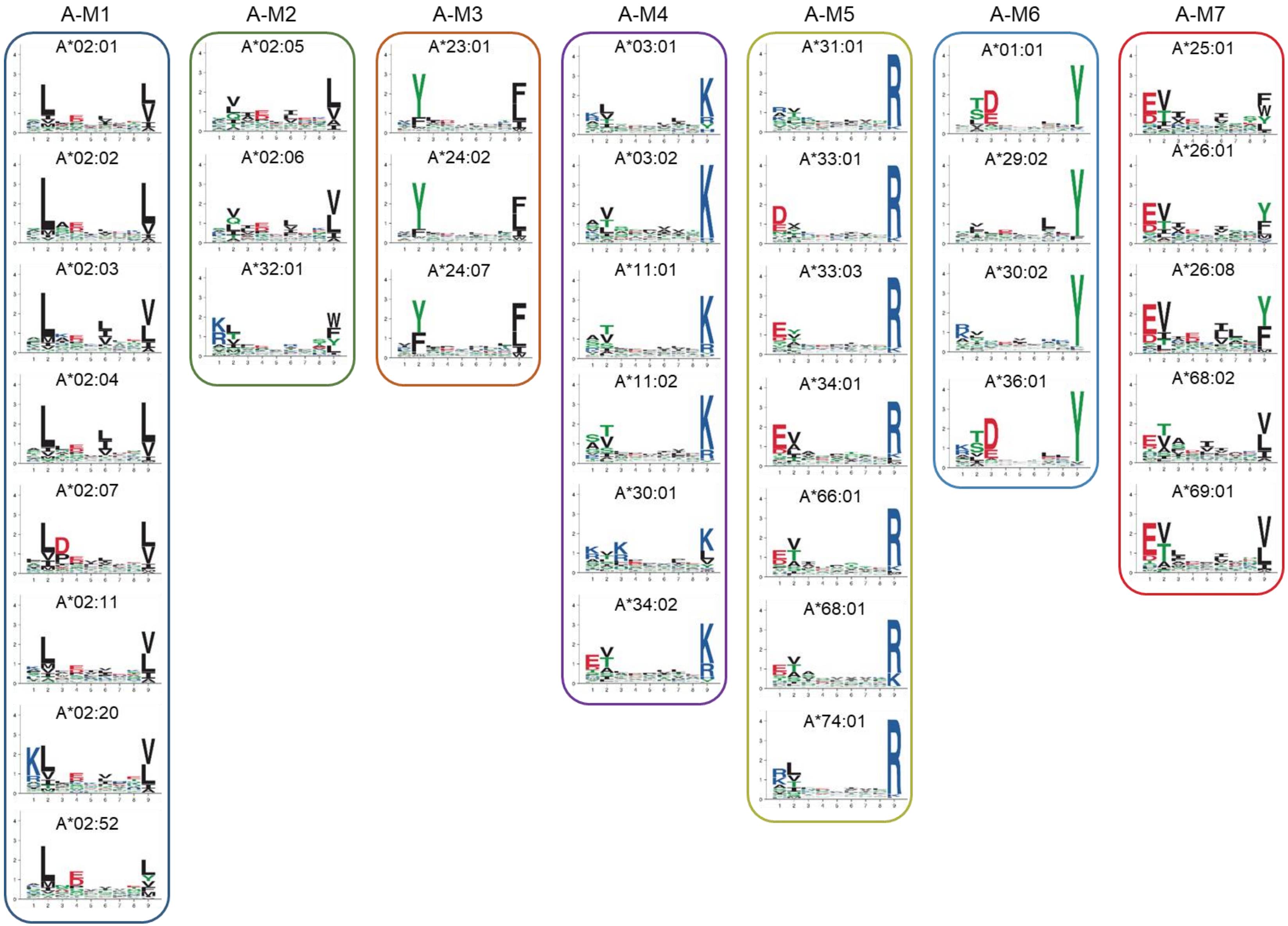

Fig. S2. Peptide motifs of 36 HLA-A allotypes according to their PBM groups. PBM of each allotype are depicted as sequence logos representing the amino acid preferences at each position of a 9-mer binding core. Logos are grouped according to the results of the hierarchical clustering shown in **Fig. 1** of the main manuscript.

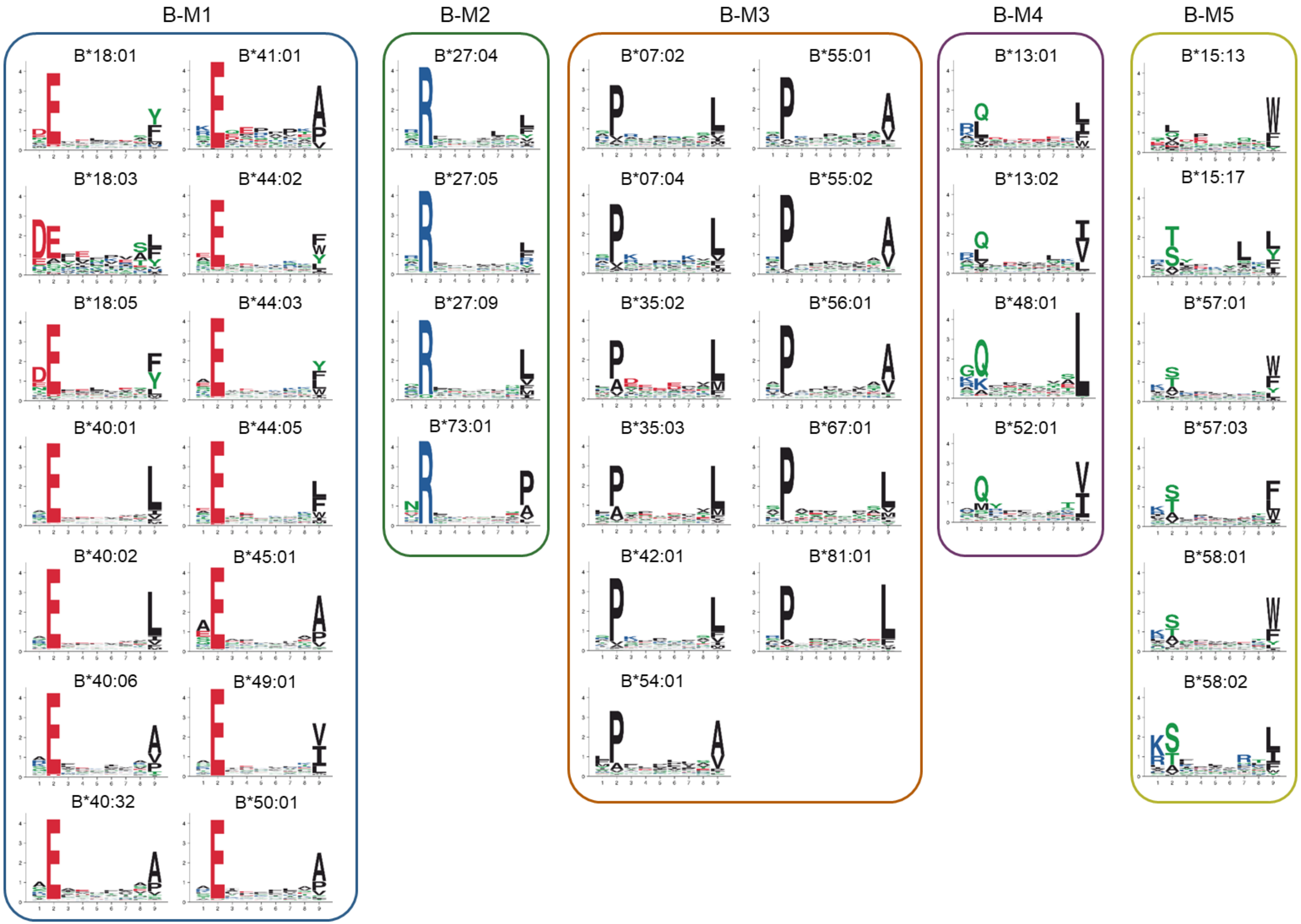

Fig. S3. Peptide motifs of 63 HLA-B allotypes according to their PBM groups. PBM of each allotype are depicted as sequence logos representing the amino acid preferences at each position of a 9-mer binding core. Logos are grouped according to the results of the hierarchical clustering shown in **Fig. 1** of the main manuscript.

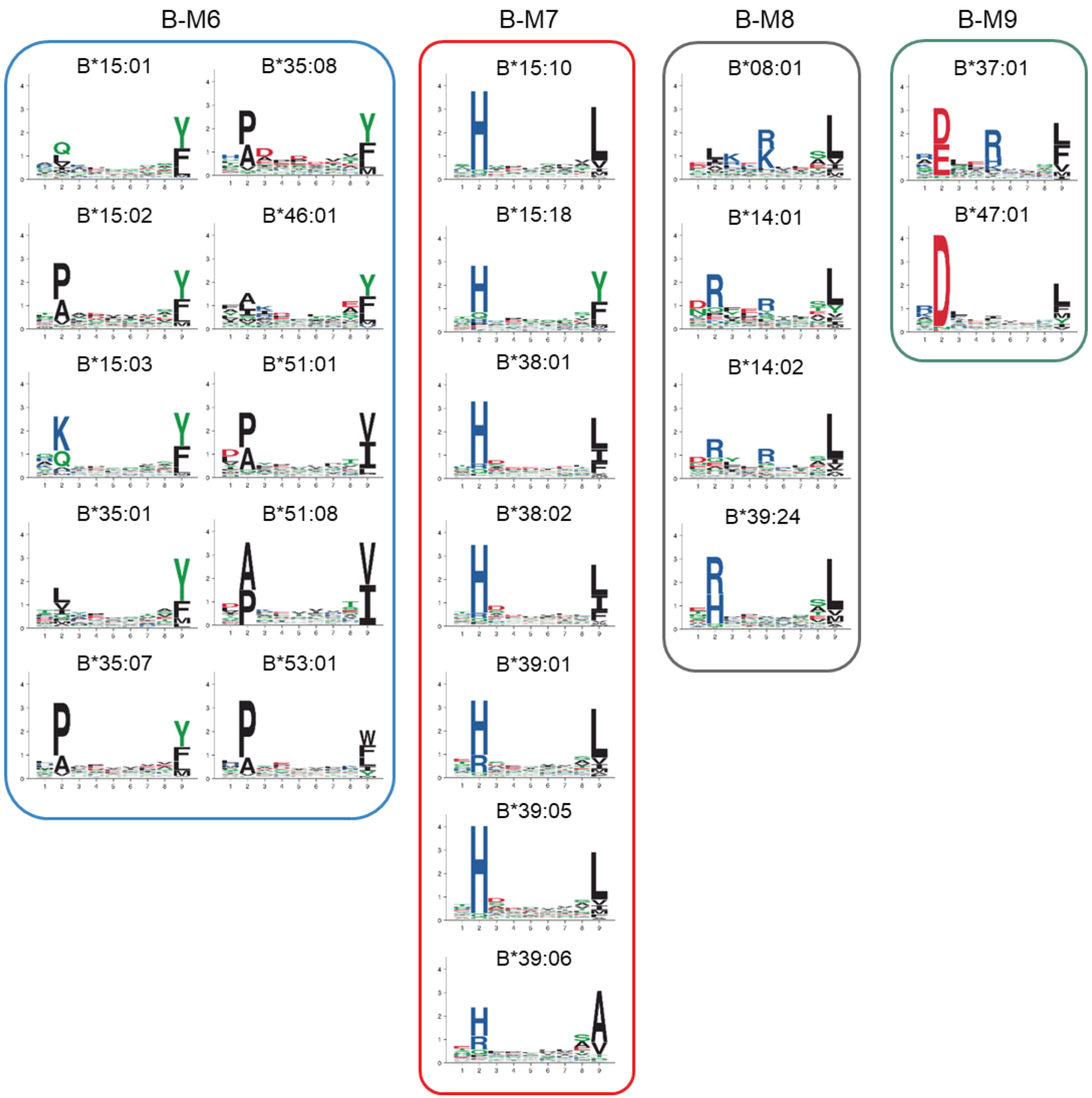

Fig. S3 (cont.). Peptide motifs of 63 HLA-B allotypes according to their PBM groups. PBM of each allotype are depicted as sequence logos representing the amino acid preferences at each position of a 9-mer binding core. Logos are grouped according to the results of the hierarchical clustering shown in **Fig. 1** of the main manuscript.

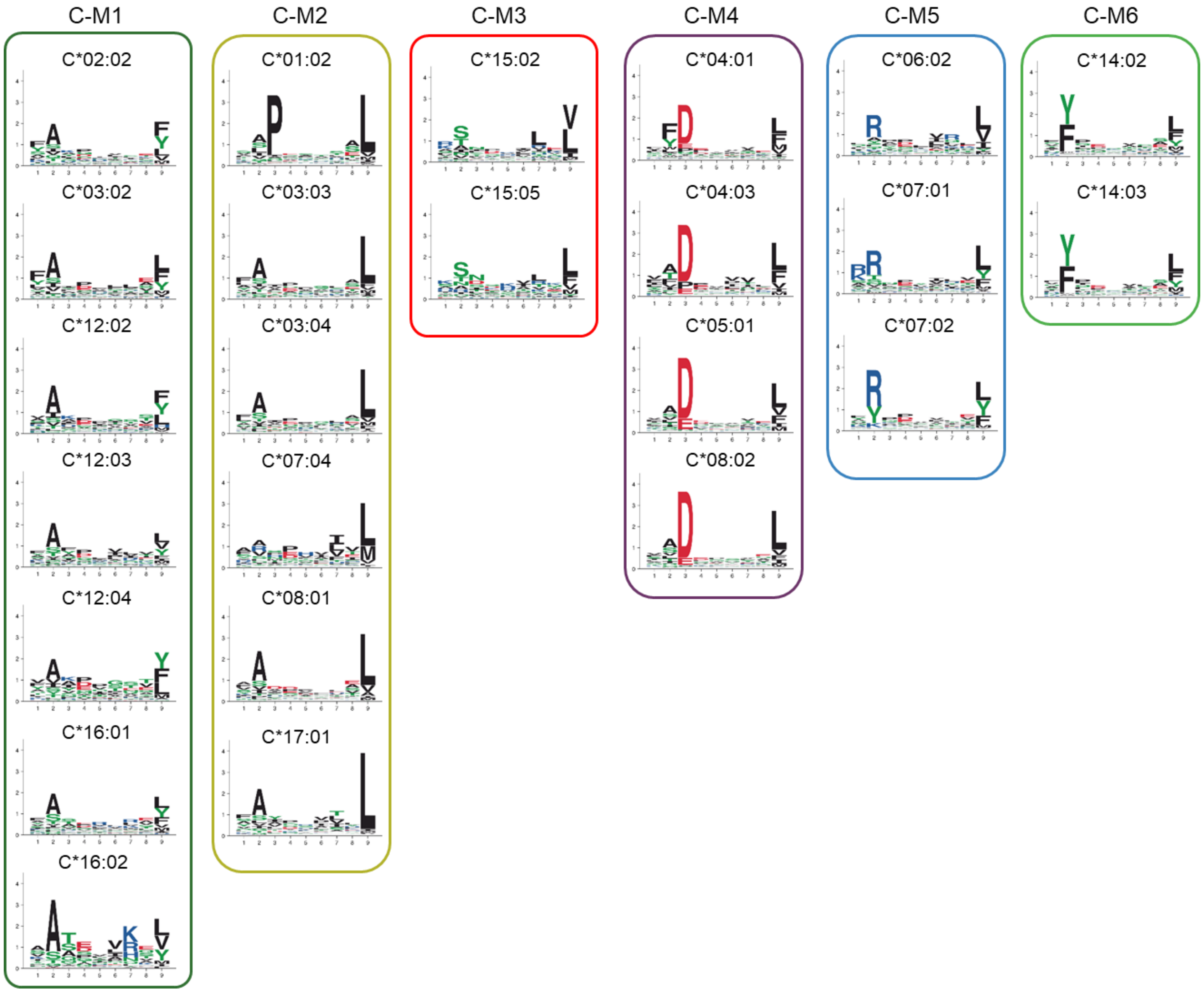

Fig. S4. Peptide motifs of 24 HLA-C allotypes according to their PBM groups. PBM of each allotype are depicted as sequence logos representing the amino acid preferences at each position of a 9-mer binding core. Logos are grouped according to the results of the hierarchical clustering shown in **Fig. 1** of the main manuscript.

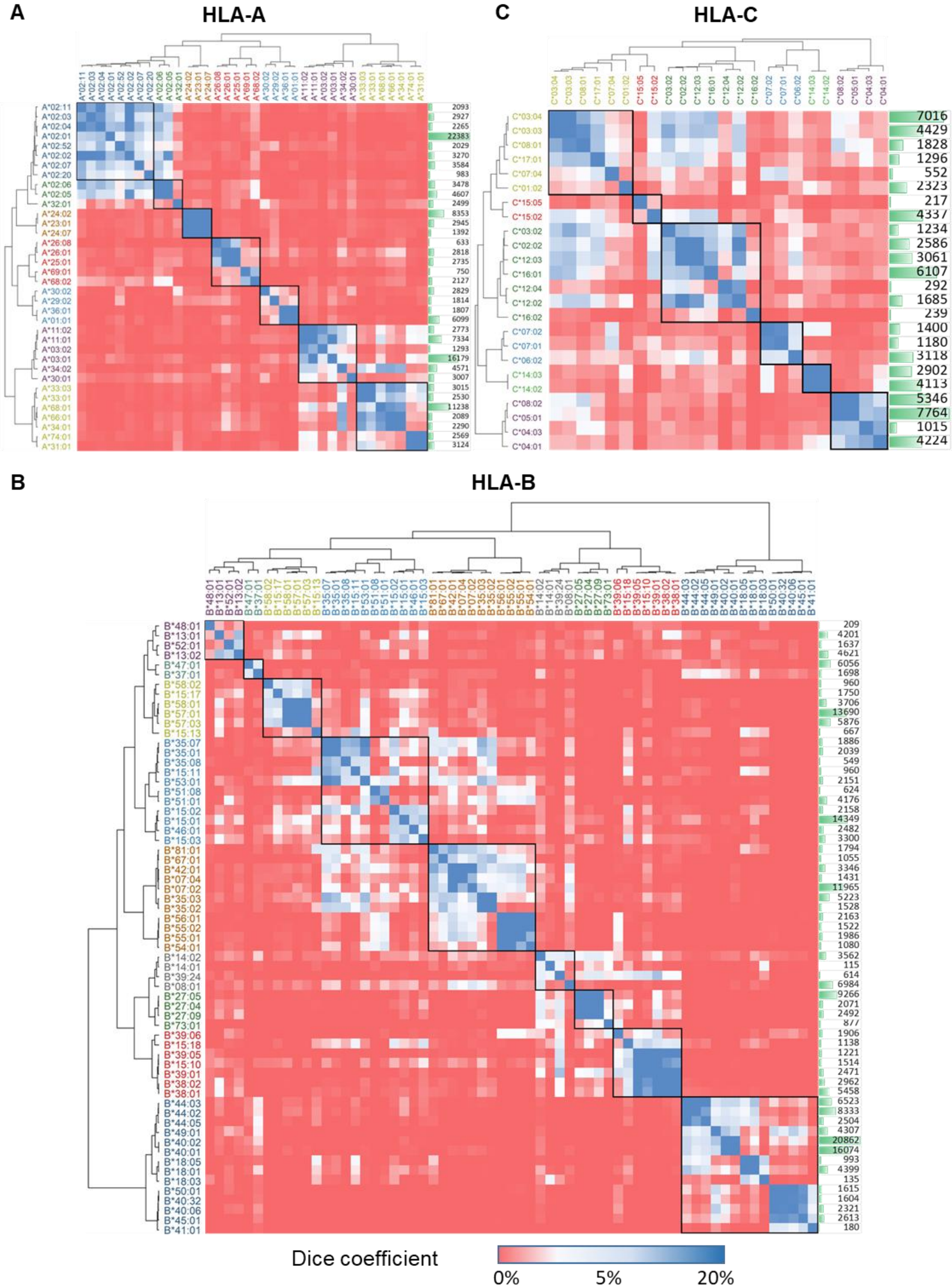

Fig. S5. Immunopeptidome overlaps of HLA class I allotypes divided by PBM groups. Heatmaps display Dice coefficients calculated for pairwise comparison of peptides from each allotype arranged according to the PBM classification reported in **Fig. 1** of the main manuscript. Boxes mark comparisons within the same PBM group. Peptide counts per allotype are indicated by bar graphs on the right side. **A** Immunopeptidome overlaps of 36 HLA-A allotypes. **B** Immunopeptidome overlaps of 63 HLA-B allotypes. **C** Immunopeptidome overlaps of 24 HLA-C allotypes.

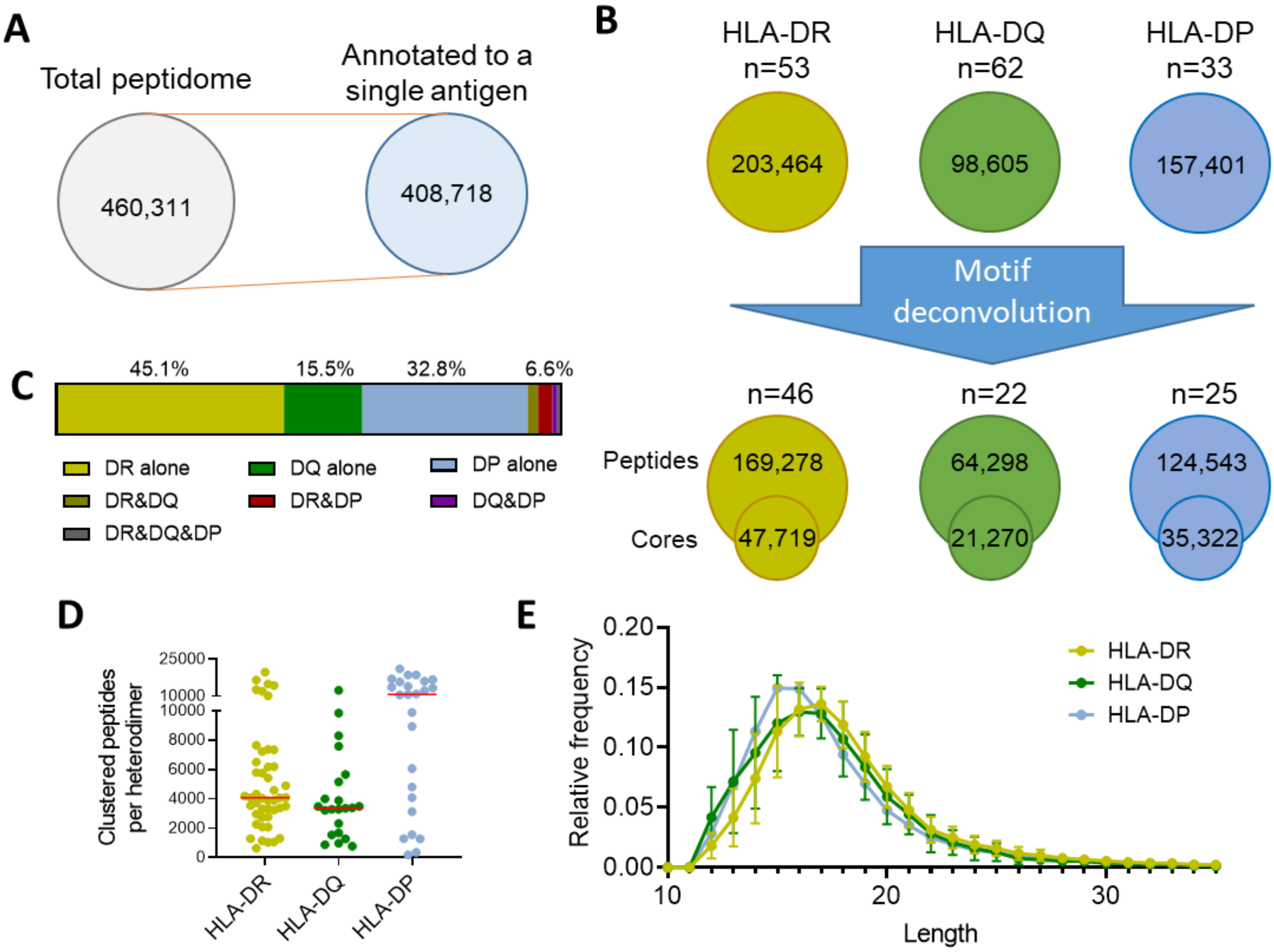

Fig. S6. Overview of the immunopeptidomics data used for the PBM identification and clustering for HLA class II allotypes. This figure summarizes key steps and metrics from the analysis of HLA class II immunopeptidomics datasets used for the PBM classification reported in **Table 2** and **Fig. 2** of the main manuscript. **A** Total number of HLA peptides collected from different immunopeptidomics studies[13-15], and subset with annotation to individual antigens. **B** Motif deconvolution further reduced the number of peptides assigned to each HLA class II allotype, resulting in a total count of 334,089 ligands. **C** Bar charts showing the fraction of peptides assigned to either HLA-DR, -DQ, or -DP molecules, or shared across loci. **D** Number of peptides with a high-quality HLA class II PBM identified per heterodimer. **E** Length of clustered peptides confirming characteristic distribution of HLA class II peptides with a median length ranging from 15 to 19 amino acids.

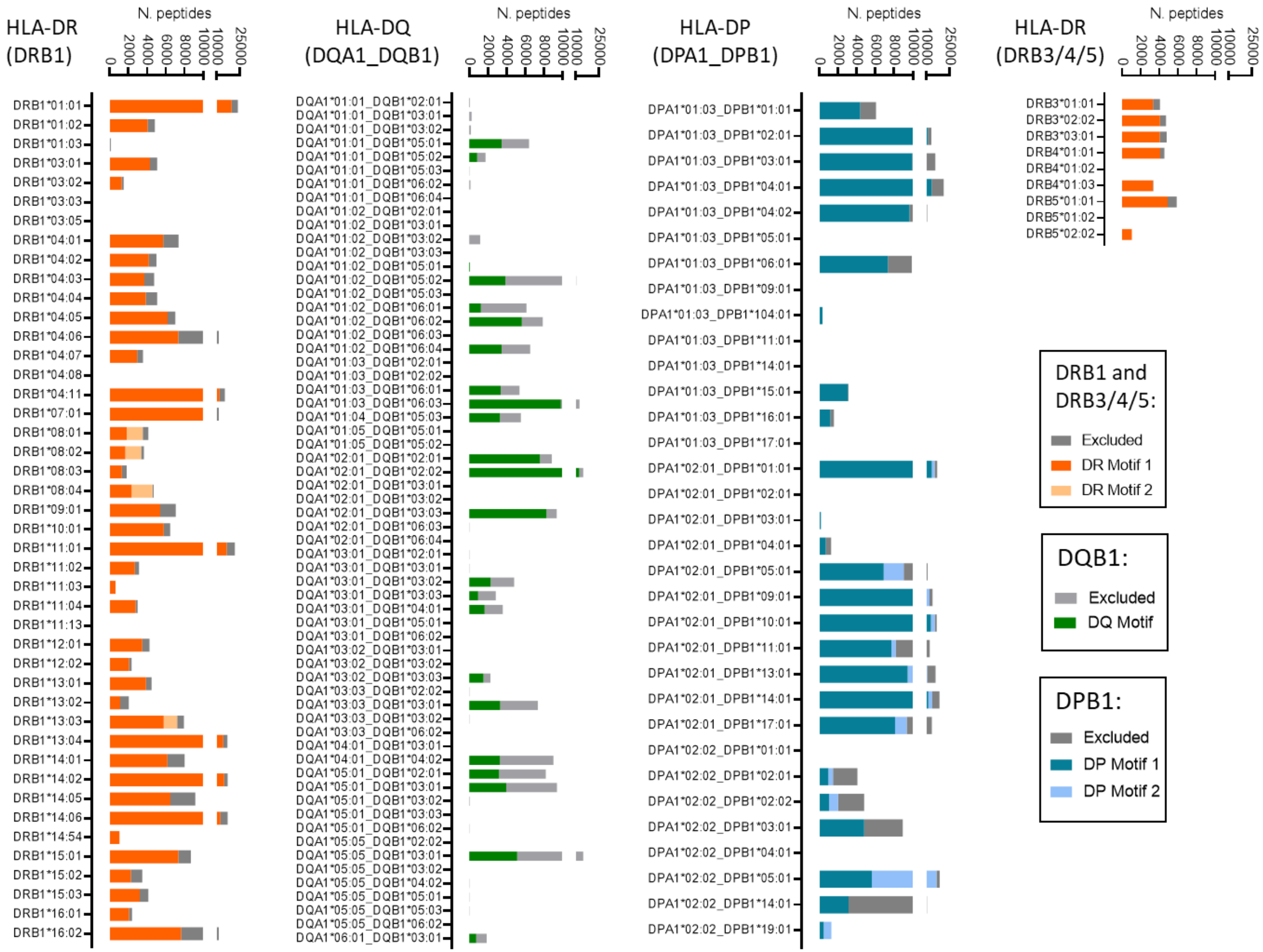

Fig. S7. Motif deconvolution results per HLA class II heterodimer across loci. Bar graphs summarize motif deconvolution output for each HLA class II heterodimer, grouped by locus: HLA-DR (in orange; including HLA-DRB1 and -DRB3/4/5 encoded heterodimers), HLA-DQ (in green), and HLA-DP (in blue). For each heterodimer, the total number of peptides analyzed and the fraction of those assigned to either a primary or secondary motif (in dark/light orange for HLA-DR, in green for HLA-DQ, and dark/light blue for HLA-DP) are indicated. In grey, the fraction of peptides excluded as potential contaminants due to lack of a detectable binding motif, or displaying motif characteristics of a different Class II locus.

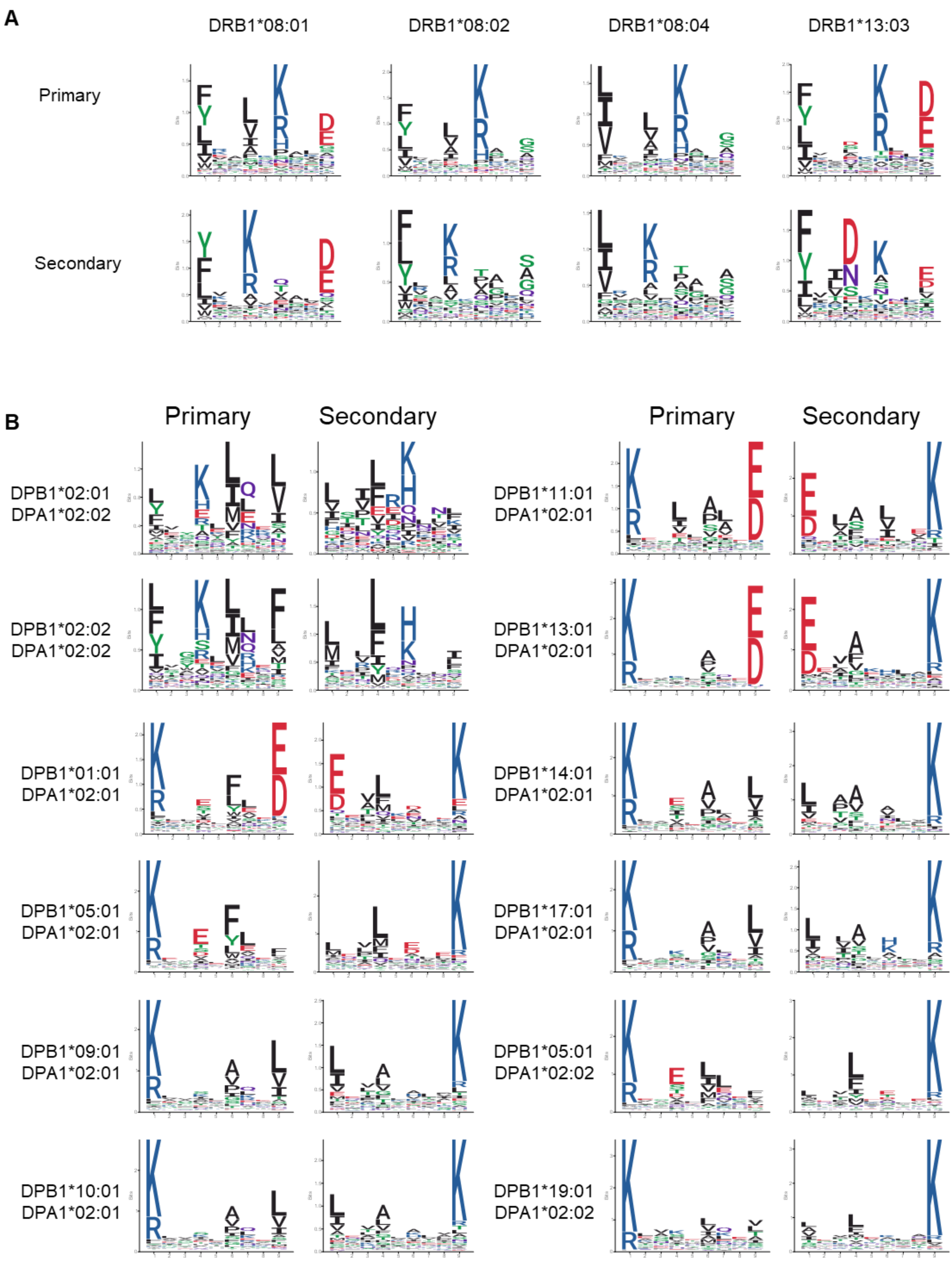

Fig. S8. Primary and secondary motifs identified in HLA-DR and HLA-DP allotypes. Sequence logos display the primary and secondary PBM derived from immunopeptidomics data for 4 HLA-DR (**A**) and 12 HLA-DP (**B**) allotypes. The logos represent amino acid preferences across a 9-residue binding core.

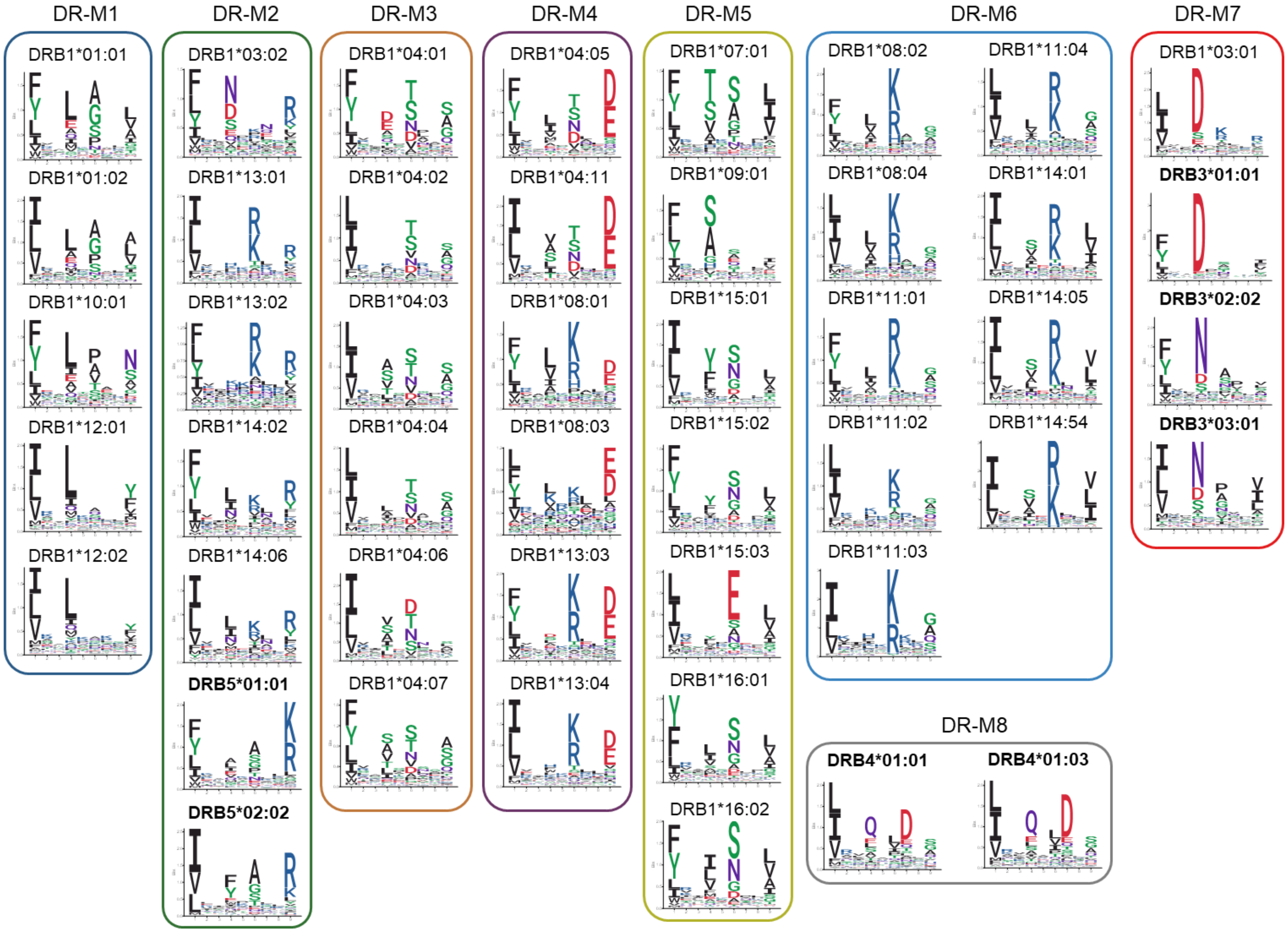

Fig. S9. Peptide motifs of 46 HLA-DR allotypes according to their PBM groups. PBM of each allotype are depicted as sequence logos representing the amino acid preferences at each position of a 9-mer binding core. Logos are grouped according to the results of the hierarchical clustering shown in **Fig. 2** of the main manuscript. In bold, allotype encoded by the paralogues DRB3/4/5.

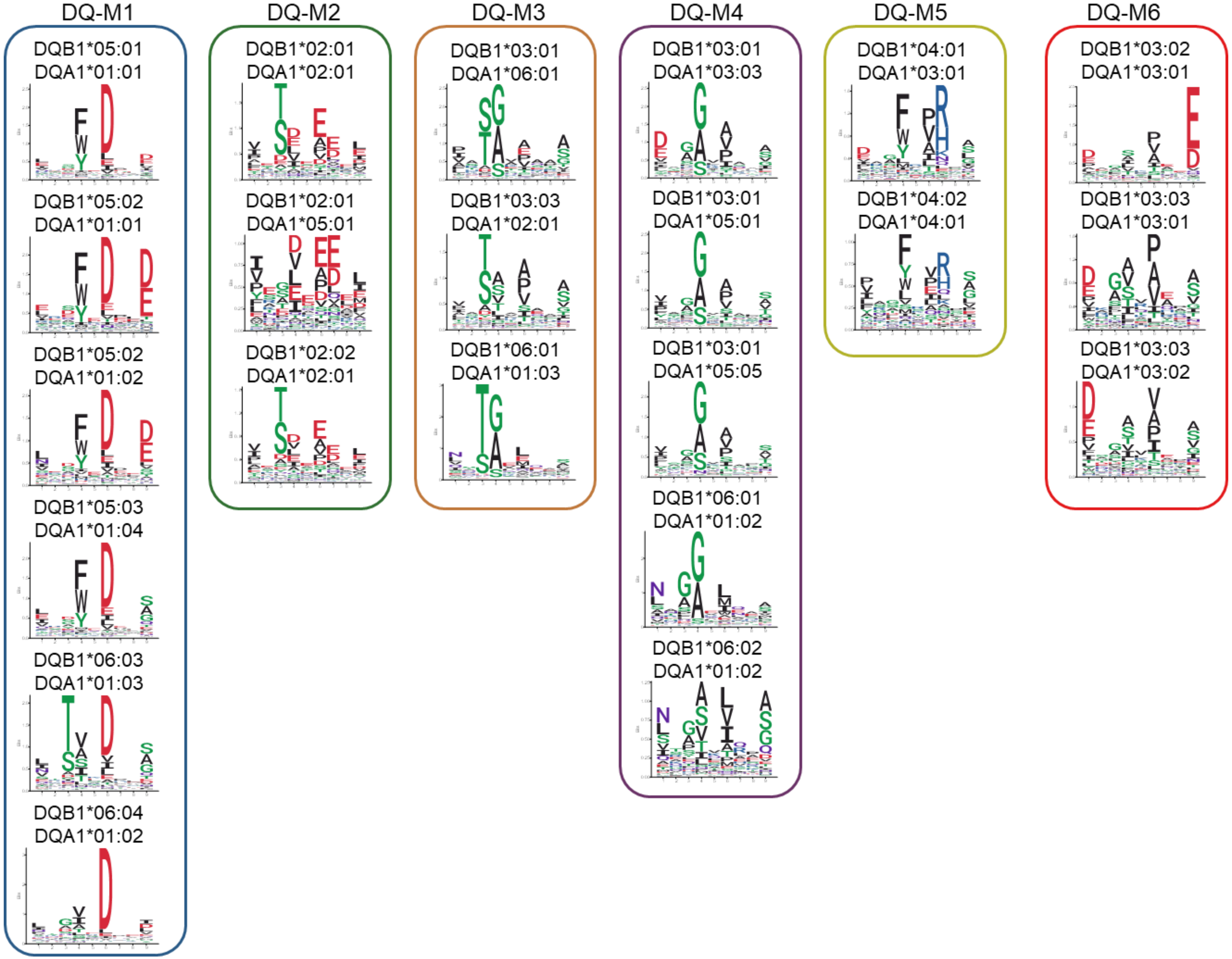

Fig. S10. Peptide motifs of 22 HLA-DQ allotypes according to their PBM groups. PBM of each allotype are depicted as sequence logos representing the amino acid preferences at each position of a 9-mer binding core. Logos are grouped according to the results of the hierarchical clustering shown in **Fig. 2** of the main manuscript.

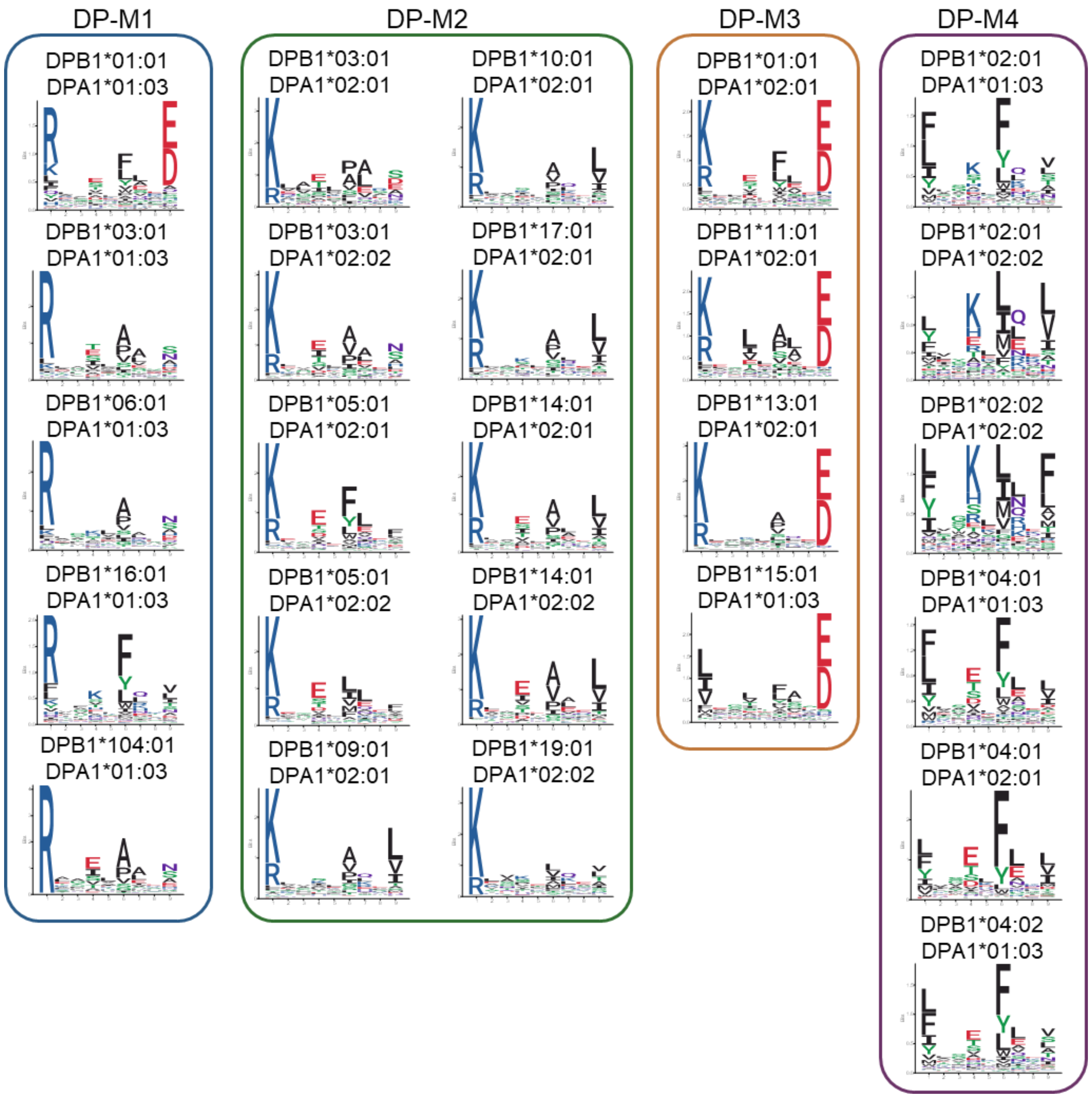

Fig. S11. Peptide motifs of 25 HLA-DP allotypes according to their PBM groups. PBM of each allotype are depicted as sequence logos representing the amino acid preferences at each position of a 9-mer binding core. Logos are grouped according to the results of the hierarchical clustering shown in **Fig. 2** of the main manuscript.

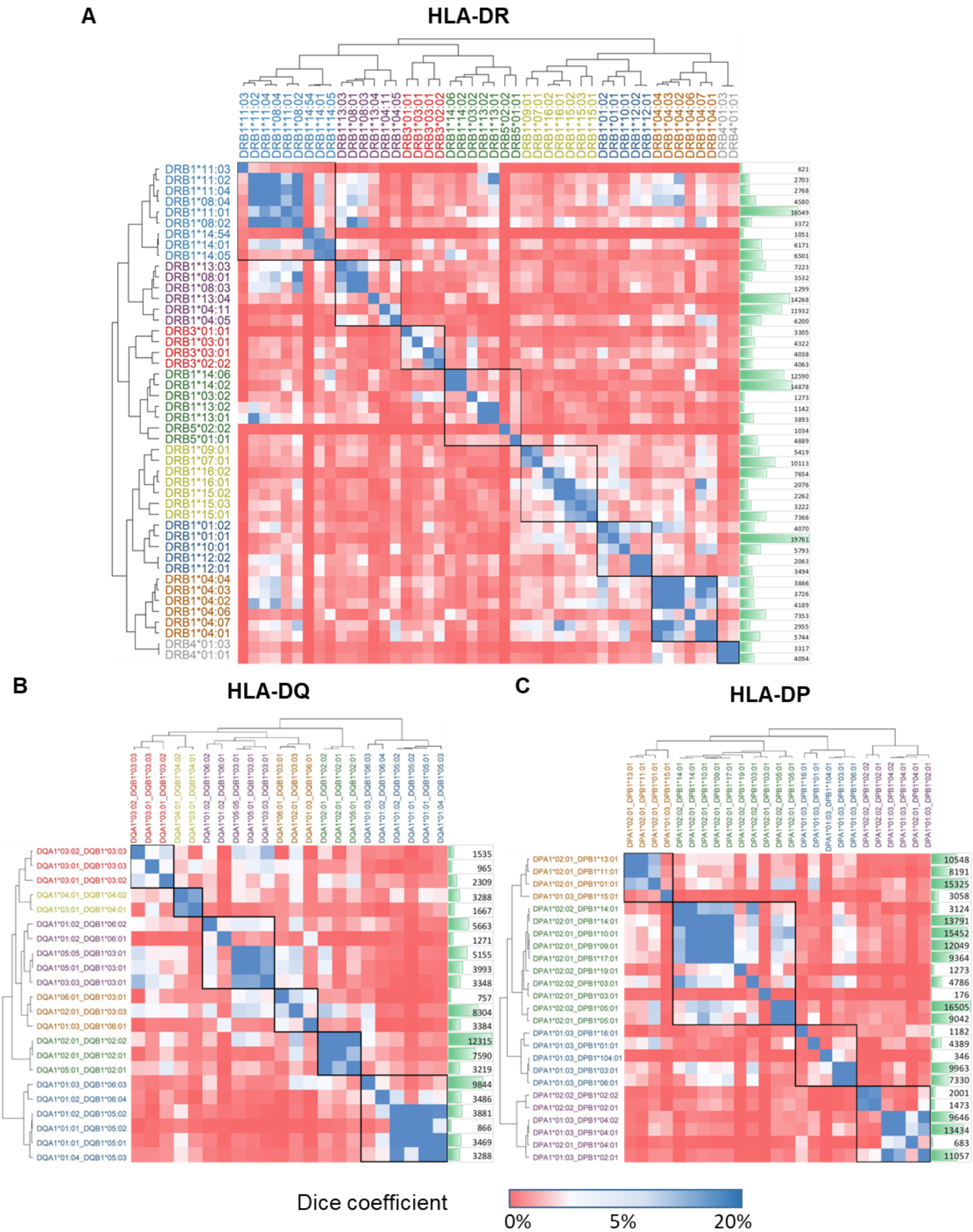

Fig. S12. Immunopeptidome overlaps of HLA class II allotypes divided by PBM groups. Heatmaps display Dice coefficients calculated for pairwise comparison of peptides from each allotype arranged according to the PBM classification reported in **Fig. 2** of the main manuscript. Boxes mark comparisons within the same PBM group. Peptide counts per allotype are indicated by bar graphs on the right side. **A** Immunopeptidome overlaps of 46 HLA-DR allotypes. **B** Immunopeptidome overlaps of 22 HLA-DQ allotypes. **C** Immunopeptidome overlaps of 25 HLA-DP allotypes.

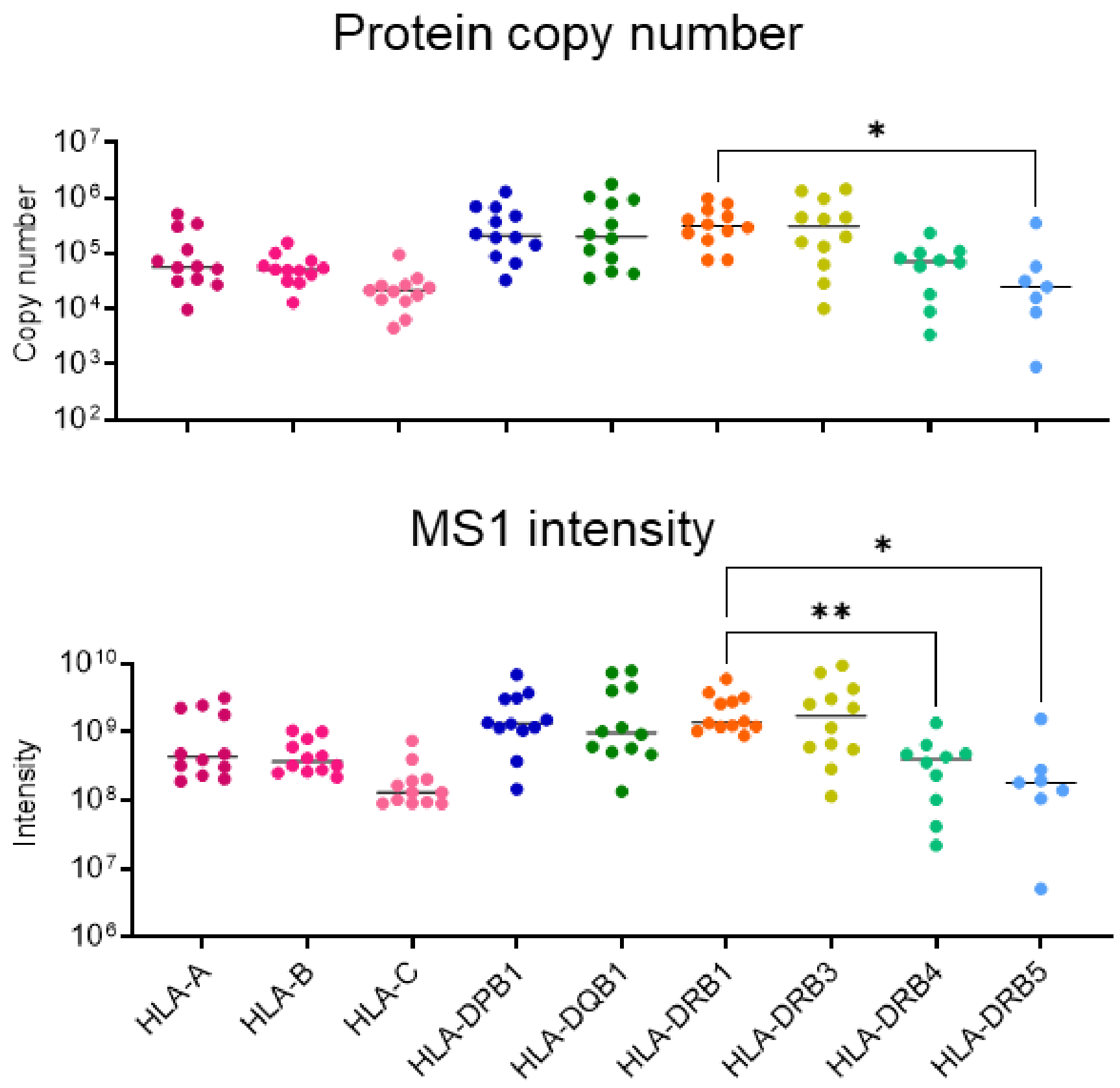

Fig. S13. Protein expression levels of HLA class I and class II molecules, including DRB3/4/5, in professional antigen-presenting cells. Dot plots represent the median values of gene expression by protein copy number (above) and MS1 intensity (below) as reported by Rieckmann et al.[26] for naïve/memory B cells, myeloid/plasmacytoid Dendritic cells, and classical monocytes at steady-state or activated, and intermediate/non-classical monocytes at steady-state (N=12, 4 replicates per cell line). Kruskal-Wallis test was performed to compare expression of HLA class II beta genes (HLA-DRB1, -DQB1, -DPB1, and -DRB3/4/5). * and ** indicate p<0.05 and <0.01, respectively.

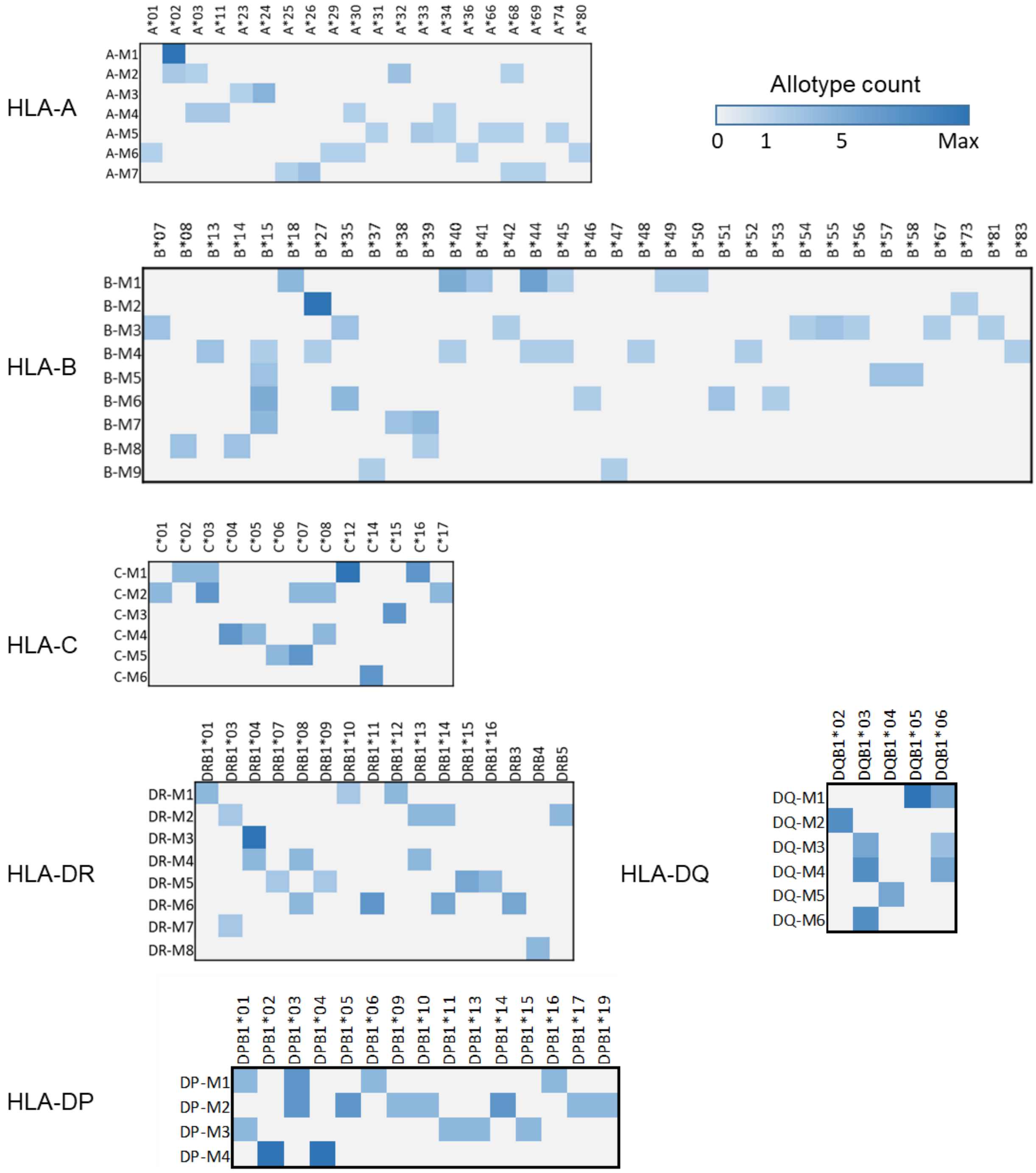

Locus	Cramér's V (95%CI)	Fisher's exact adj. P value	NMI
HLA-A	0.89 (0.86-0.97)	<0.001	0.73
HLA-B	0.88 (0.84-0.94)	<0.001	0.67
HLA-C	0.92 (0.86-1.00)	<0.001	0.72
HLA-DRB1	0.80 (0.79-0.92)	<0.001	0.70
HLA-DQB1	0.84 (0.76-0.93)	<0.001	0.68
HLA-DPB1	0.89 (0.82-1.00)	0.0046	0.60

Fig. S14. Contingency tables of HLA allotypes by PBM groups and first-field allele groups. For each locus, contingency tables display the distribution of allotypes across PBM groups on the left and first-field allele groups on the top. A summary table reports Cramér's V coefficients with bootstrapped 95% confidence intervals (measuring the strength of association between the two variables), Fisher exact test p-values adjusted using the Benjamini–Hochberg procedure, and normalized mutual information (NMI) scores indicating the proportion of shared information. High Cramér's V values and moderate NMI scores (>0.6), indicate strong but incomplete correspondence.

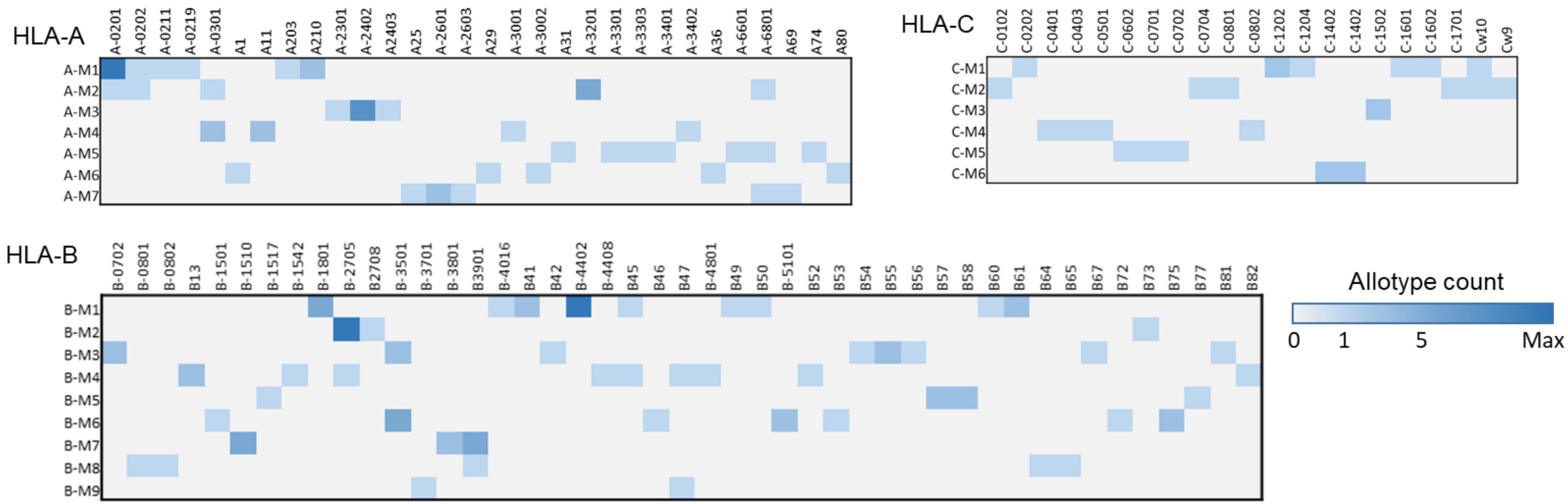

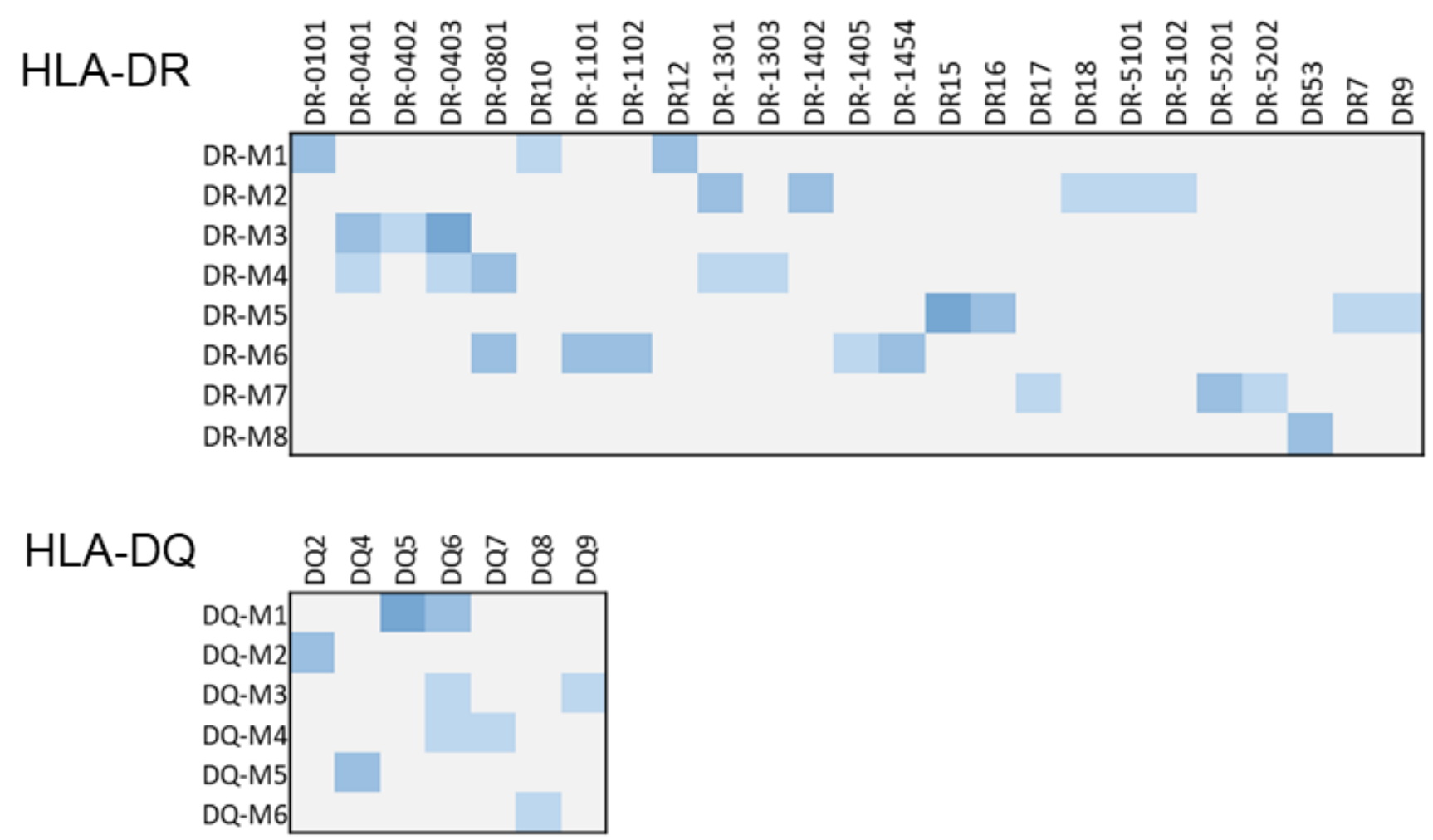

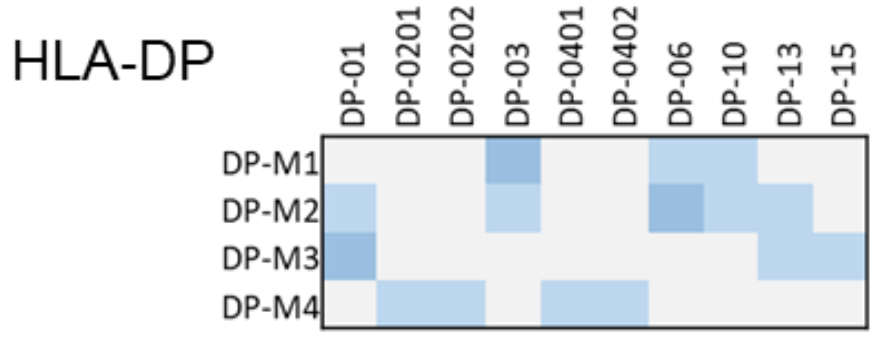

Locus	Cramér's V (95%CI)	Fisher's exact adj. P value	NMI
HLA-A	0.93 (0.89-1.00)	<0.001	0.70
HLA-B	0.93 (0.90-0.99)	<0.001	0.71
HLA-C	0.98 (0.96-1.00)	<0.001	0.73
HLA-DRB1	0.93 (0.90-0.99)	<0.001	0.73
HLA-DQB1	0.90 (0.78-1.00)	<0.001	0.79
HLA-DPB1	0.76 (0.73-0.95)	0.4006	0.52

Fig. S15. Contingency tables of HLA allotypes by PBM groups and serological groups. For each locus, contingency tables display the distribution of allotypes across PBM groups on the left and serological antigen groups[19] listed on the top. Summary tables report Cramér's V coefficients with bootstrapped 95% confidence intervals (measuring the strength of association between the two variables), Fisher exact test p-values adjusted using the Benjamini–Hochberg procedure, and normalized mutual information (NMI) scores indicating the proportion of shared information. High Cramér's V values and moderate NMI scores (>0.6), indicate strong but incomplete correspondence between PBM and antigen groups.

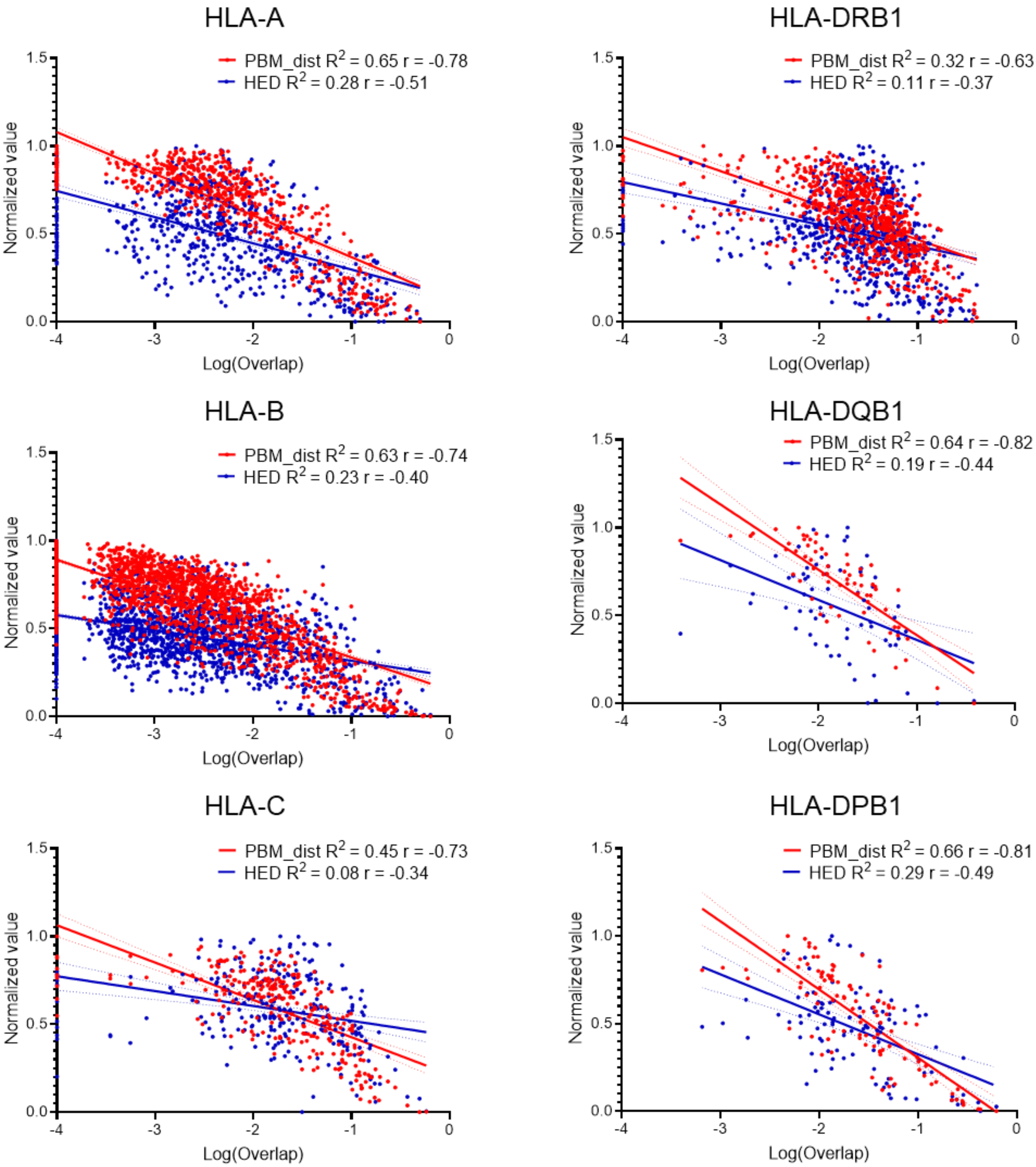

Fig. S16. Correlation between immunopeptidome overlaps and HED or PBM distance. Scatter plots comparing immunopeptidome overlaps between pairs of HLA allotypes (measured by Dice coefficient at the X-axis) and two distance metrics (Y-axis): HED (shown in blue) and PBM distance calculated as the 1 minus Pearson correlation of corresponding position weight matrices (shown in red). Each dot represents a pair of HLA allotypes in each of the 6 loci. In total, the number of allotype pairs tested for HLA-A, -B, -C, -DR, DQ and -DP were 630, 1953, 276, 741, 66 and 105, respectively. Correlations are assessed using both Pearson's R^2 and Spearman's r coefficients. Trend lines with 95% confidence intervals (dotted lines) are also indicated.
